# Cohesin’s ATPase Activity Couples Cohesin Loading onto DNA with Smc3 Acetylation

**DOI:** 10.1016/j.cub.2014.08.011

**Published:** 2014-10-06

**Authors:** Rene Ladurner, Venugopal Bhaskara, Pim J. Huis in ’t Veld, Iain F. Davidson, Emanuel Kreidl, Georg Petzold, Jan-Michael Peters

**Affiliations:** 1 Research Institute of Molecular Pathology, Dr. Bohr-Gasse 7, 1030 Vienna, Austria

## Abstract

**Background:**

Cohesin mediates sister chromatid cohesion by topologically entrapping sister DNA molecules inside its ring structure. Cohesin is loaded onto DNA by the Scc2/NIPBL-Scc4/MAU2-loading complex in a manner that depends on the adenosine triphosphatase (ATPase) activity of cohesin’s Smc1 and Smc3 subunits. Subsequent cohesion establishment during DNA replication depends on Smc3 acetylation by Esco1 and Esco2 and on recruitment of sororin, which “locks” cohesin on DNA by inactivating the cohesin release factor Wapl.

**Results:**

Human cohesin ATPase mutants associate transiently with DNA in a manner that depends on the loading complex but cannot be stabilized on chromatin by depletion of Wapl. These mutants cannot be acetylated, fail to interact with sororin, and do not mediate cohesion. The absence of Smc3 acetylation in the ATPase mutants is not a consequence of their transient association with DNA but is directly caused by their inability to hydrolyze ATP because acetylation of wild-type cohesin also depends on ATP hydrolysis.

**Conclusions:**

Our data indicate that cohesion establishment involves the following steps. First, cohesin transiently associates with DNA in a manner that depends on the loading complex. Subsequently, ATP hydrolysis by cohesin leads to entrapment of DNA and converts Smc3 into a state that can be acetylated. Finally, Smc3 acetylation leads to recruitment of sororin, inhibition of Wapl, and stabilization of cohesin on DNA. Our finding that cohesin’s ATPase activity is required for both cohesin loading and Smc3 acetylation raises the possibility that cohesion establishment is directly coupled to the reaction in which cohesin entraps DNA.

## Highlights

• Cohesin initially interacts with chromatin transiently via the loading complex
• Cohesin’s ATPase activity is required for dynamic chromatin-cohesin interactions
• Smc3 acetylation does not significantly alter cohesin’s ATPase activity
• Cohesin’s ATPase activity is required for Smc3 acetylation

## Graphical Abstract

**Figure:**
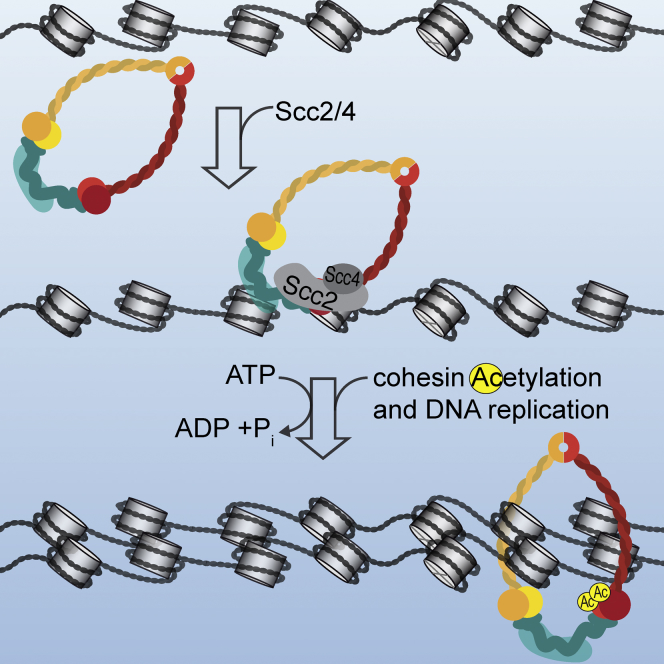

## Teaser

Ladurner et al. present evidence that the adenosine triphosphatase (ATPase) activity of the cohesin complex is required for cohesin acetylation, suggesting that sister-chromatid entrapment by cohesin is coupled to the establishment of cohesion.

## Introduction

**Figure 1:**
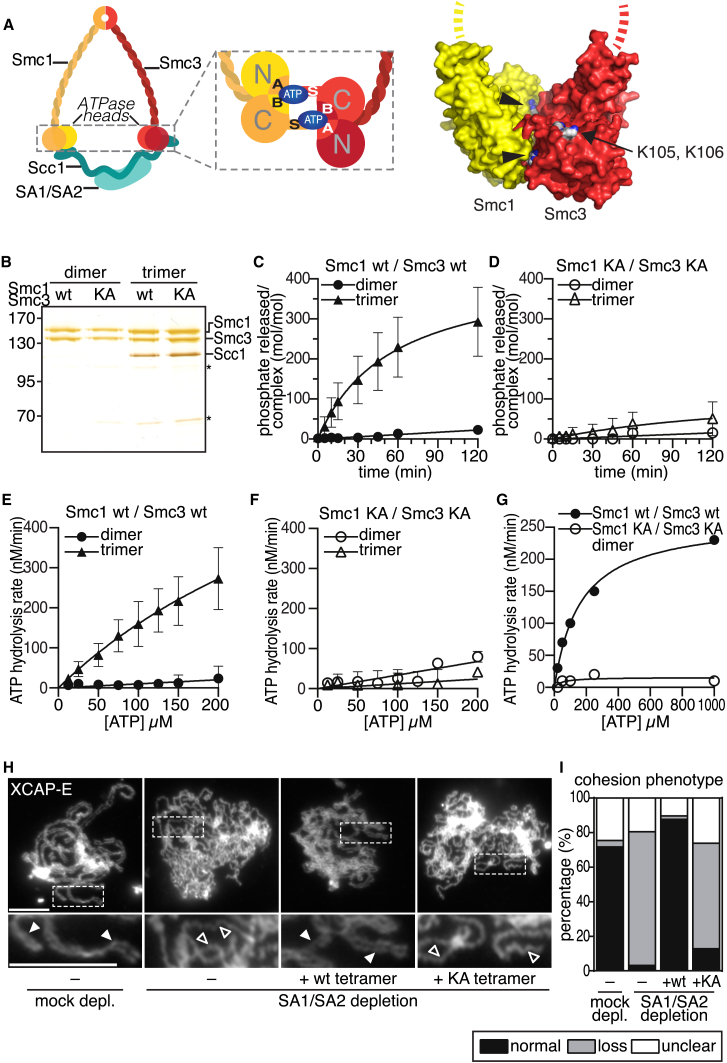
Reconstitution and Functional Characterization of Human Cohesin (A) Schematic model of a cohesin tetramer. Enlarged view indicates how Walker A (A), signature (S), and Walker B (B) motifs cooperate to bind and hydrolyze ATP. The model structure on the right shows the proximity between Smc3 acetylation (K105 and K106; arrow) and ATP binding sites (arrowheads) (based on Protein Data Bank 1W1W; [3]). (B) Purified WT and Walker A mutant (KA) dimeric and trimeric (with Scc1) cohesin complexes were analyzed by silver staining. (C and D) Time course quantification of phosphate released after incubation of purified complexes with ATP [γ-^32^P] to determine specific enzymatic activities. Error bars denote SD (n = 3). (E and F) Substrate dose-response quantification of purified complexes to measure ATP hydrolysis rates. Error bars denote SD (n = 3). (G) Substrate dose response of cohesin dimers at high enzyme concentration to quantify ATP hydrolysis rates. (H) XCAP-E staining of *Xenopus* extracts after depletion (depl.) of SA1/SA2, addition of purified human cohesin tetramers, and assembly of mitotic chromosomes to determine the degree of sister chromatid cohesion. Closed arrowheads indicate paired, open arrowheads unpaired sister chromatids. Scale bars represent 10 μm. The efficiency of cohesin depletion in this experiment is shown in Figure 5A. (I) Chromosome cohesion phenotype after XCAP-E staining as depicted in (H) was quantified (n ≥ 155 per condition). See also Figure S1.

During DNA replication, newly synthesized DNA molecules become physically connected with each other. This sister chromatid cohesion enables the biorientation of chromosomes on the mitotic spindle and is therefore essential for proper chromosome segregation [1]. Cohesion is mediated by the ring-shaped cohesin complex (reviewed in [2]), which contains a heterodimer of the highly elongated Smc1 and Smc3 proteins. Both of these contain long intramolecular coiled coils, a “hinge” region at their central folds, and a nucleotide-binding domain (NBD), which is jointly formed by their N and C termini (Figure 1A). Smc1 and Smc3 dimerize via their hinge domains, whereas association of their NBDs results in the formation of an ABC transporter-like adenosine triphosphatase (ATPase) domain that can bind and hydrolyze two ATP molecules. The “kleisin” subunit Scc1 (also known as Rad21 or Mcd1) bridges the NBDs of Smc1 and Smc3, resulting in a tripartite ring structure. Scc1 is associated with a fourth subunit, called Scc3 in yeast, which in somatic vertebrate cells exists in two isoforms: SA1 and SA2.

Cohesin mediates cohesion by entrapping sister chromatids inside its ring structure [4]. Chromatin fibers have been proposed to enter the cohesin ring via an “entry gate” that is thought to be located between the hinge regions of Smc1 and Smc3 [5, 6]. The loading of cohesin onto chromatin requires cohesin’s ATPase activity [7, 8] and a separate loading complex, consisting of the proteins Scc2/NIPBL and Scc4/MAU2 [9, 10]. Experiments in yeast have shown that cohesin complexes deficient in ATP hydrolysis associate with chromatin in a Scc2-dependent but transient manner [11], whereas topological loading of cohesin onto DNA in vitro is stimulated by the loading complex and depends on cohesin’s ATPase activity [10]. These observations suggest that the loading complex targets cohesin to chromatin, whereas the ATPase reaction mediates entrapment of DNA inside the cohesin ring.

Once properly loaded, cohesin can be released from DNA by disengagement of the Smc3-Scc1 interface [5, 12, 13]. The opening of this “exit gate” is under the control of Scc3 and the cohesin-interacting proteins Pds5 and Wapl [12, 14]. Cohesin release via the exit gate is thought to contribute to dynamic noncanonical functions of the complex, such as regulation of chromatin structure and gene expression (discussed in [12, 15]), and in vertebrates is used to remove cohesin from chromosome arms in early mitosis [5, 16, 17].

To be able to mediate sister chromatid cohesion, cohesin’s exit gate has to be “locked” to prevent precocious release of cohesin from chromatin. To achieve this, the Smc3 subunit is acetylated during DNA replication on two conserved Lys residues (K105,106 in vertebrates) by Eco1 proteins [18, 19, 20]. In vertebrates, two Eco1 orthologs exist: Esco2, which is expressed during DNA replication, and Esco1, which is present throughout the cell cycle [21]. Smc3 acetylation during DNA replication leads to the association of cohesin with sororin [22, 23], a protein that inhibits Wapl and thereby prevents opening of the exit gate [5, 12, 13, 23]. Sororin is essential for cohesion in vertebrates and *D. melanogaster* [23, 24], but no sororin ortholog has yet been identified in fungi. In these organisms, cohesin acetylation has been proposed to prevent cohesin release by modulating cohesin’s ATPase activity [25] or through interactions between Smc3 and Wapl [26].

Although cohesion can only be established during S phase [27], cohesin is loaded onto DNA already before DNA replication [28], presumably by entrapping a single chromatid fiber. It has been proposed that cohesion can be established by such “preloaded” cohesin complexes [29], implying that DNA polymerases are able to move through cohesin rings. This hypothesis could explain how DNA replication would automatically lead to entrapment of sister chromatids inside cohesin’s ring structure, but direct evidence for this “replication-through-the-ring” model is missing.

To understand how cohesin is loaded onto DNA and establishes cohesion, we have generated human cohesin complexes in wild-type (WT), nonacetylatable, and ATP hydrolysis-deficient forms and have analyzed their properties as purified complexes, in *Xenopus* egg extracts, and by expression in HeLa cells. The results from these experiments provide further support for the hypothesis [10, 11] that cohesin loading occurs in two steps in which cohesin is first recruited to DNA by the loading complex and subsequently entraps DNA in an ATPase-dependent manner. Unlike previously proposed [25], cohesin’s ATPase activity is not detectably altered by Smc3 acetylation. Unexpectedly, however, the opposite is the case. Smc3 acetylation is strictly dependent on cohesin’s ability to hydrolyze ATP, both in vivo and in vitro. Because ATP hydrolysis is essential for entrapment of DNA inside the cohesin ring, our results indicate that cohesin acetylation is coupled to the loading of cohesin onto DNA. We discuss the implications of this finding, namely that cohesion establishment during DNA replication may not only depend on Smc3 acetylation but also on de novo loading of cohesin onto DNA.

## Results

### Recombinant Human Cohesin Complexes Are Functional ATPases

To guide the generation of human cohesin mutants, we performed in silico modeling of the human Smc1 and Smc3 ATPase domain using a yeast Smc1 crystal structure as a template [3]. As expected, the resulting model indicated that the signature motif of Smc1 contacts the Walker A and Walker B motifs of Smc3 and vice versa to form two composite ATP binding sites. At these sites, the Walker A and Walker B motifs are predicted to be required for ATP binding and hydrolysis, respectively [7]. The model also confirmed [18, 19] that these sites are in proximity to Smc3’s acetylation sites K105 and K106 (Figure 1A).

To characterize the ATPase activity of human cohesin, we expressed dimeric (Smc1-Smc3) and trimeric (Smc1-Smc3-Scc1) complexes in Baculovirus-infected insect cells, purified these complexes by tandem affinity purification, and analyzed their composition by SDS-PAGE and silver staining (Figure 1B). ATPase thin-layer chromatography assays revealed that over time, trimeric complexes hydrolyzed more ATP than dimeric complexes (Figure 1C), consistent with the previous observation that the C-terminal winged helix of Scc1 stimulated the ATPase activity of yeast dimeric cohesin [30]. In both complexes, ATP hydrolysis was caused by Smc1 and Smc3 because mutation of K38 in the Walker A motif of Smc1 and Smc3 to alanine (“KA”) reduced ATP hydrolysis (Figure 1D; Figure S1A available online).

At a concentration of 50 μM ATP, WT trimers hydrolyzed 2.4 ± 0.7 mol ATP per mol cohesin complex per minute. To measure kinetic constants, we performed substrate titration experiments with WT forms and KA mutants, obtained from three independent purifications (Figures 1E and 1F), and found that WT trimers exhibited a Michaelis-Menten constant (K_M_) of 467 ± 55 μM. When incubating trimeric cohesin with an ATP concentration close to this value, we consequently observed that the ATP hydrolysis rate increased by one order of magnitude (Figure S1B).

The observed ATP hydrolysis rate of the Smc1-Smc3 dimer was indistinguishable from “background” levels at low enzyme concentrations but could be well distinguished at concentrations above 200 nM (Figure S1C). We therefore used a 5-fold higher concentration for the dimer than for the trimer in a substrate titration experiment. Under these conditions, WT dimers hydrolyzed 0.3 mol ATP per mol complex per minute (Figures 1G and S1B). The specific activities of the human dimers and trimers are similar to the ones reported for the corresponding yeast complexes (2.1 moles of ATP per mol complex per minute for budding yeast cohesin [30]; 3.7 min^−1^ for fission yeast cohesin [10]).

To test whether the ATPase activity of human cohesin is required for cohesion, we used a *Xenopus* egg extract system. We immunodepleted endogenous *Xenopus* cohesin from interphase extract with SA1 and SA2 antibodies, added purified human tetrameric cohesin (Smc1-Smc3-Scc1-SA1; Figures S1D and S1E), allowed the extract to replicate sperm chromatin, and then triggered chromosome condensation by addition of nondegradable cyclin B. Immunofluorescence microscopy revealed that normal sister chromatid cohesion was observed in 70% of chromosome spreads from control-depleted extracts, whereas cohesion defects were observed in 80% of chromosome spreads following depletion of endogenous cohesin (Figure 1H). Adding back WT, but not KA mutant, cohesin prevented these cohesion defects (Figures 1H and 1I). These results show that the recombinant cohesin complexes generated here are able to mediate cohesion and indicate that their ATPase activity is essential for this function.

### Cohesin ATPase Mutants Associate Transiently with Chromatin

**Figure 2:**
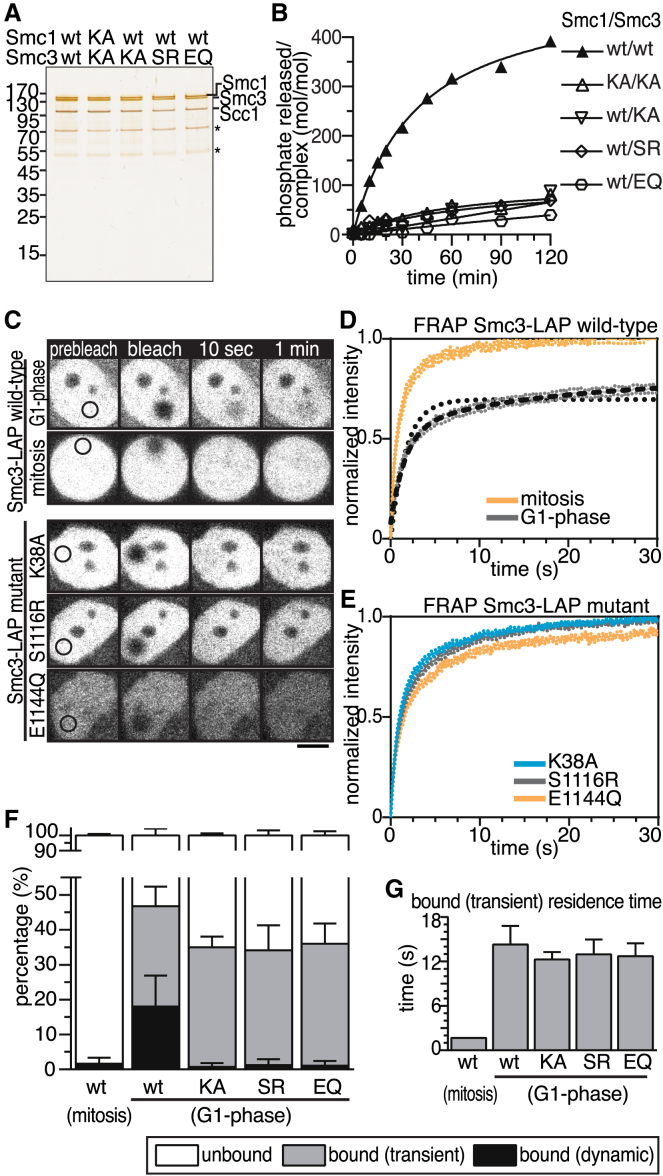
Cohesin ATPase Mutants Associate Transiently with Chromatin (A) Silver staining of purified trimeric cohesin complexes after mutation of ATPase sites in Smc3 or Smc1/Smc3 subunits (KA, Walker A mutant; SR, signature mutant; EQ, Walker B mutant). Asterisks denote unidentified proteins. (B) Time course quantification of phosphate released from ATPase reactions with cohesin mutated at one or both ATPase-active sites. (C) Still images of FRAP experiments with HeLa cells expressing mouse Smc3-LAP. Cells were synchronized in G1 phase or were in mitosis. Black circles illustrate site of bleaching (2 μm radius). Scale bar represents 10 μm. (D) Quantification of fluorescent signals after normalization from Smc3-LAP WT cells treated as in (C). Dotted line illustrates fitted curve using single-phase association; dashed line shows fitted curve with a two-phase association function. (E) Quantification of ATPase mutant FRAP signals. (F) Quantification of the cohesin distribution within the nucleus as freely diffusing (unbound), transiently chromatin-bound, and dynamically chromatin-bound populations. (G) Calculated residence time of transiently chromatin-bound cohesin pools. Error bars in (D)–(G) denote SEM (n ≥ 20 per condition in G1 phase, n = 10 in mitosis). See also Figure S2.

Because the function of cohesin’s ATPase activity has so far only been analyzed in yeast, we analyzed the properties of cohesin ATPase mutants in human cells. We first tested if point mutations in the Walker A (K38A or KA), signature motif (S1116R or SR), or Walker B (E1144Q or EQ) motifs of Smc3 are sufficient to abolish cohesin’s ATPase activity, as is the case for yeast cohesin [30]. For this purpose, we purified cohesin trimers containing Smc1, Scc1, and either WT or mutated forms of Smc3 from Baculovirus-infected insect cells (Figure 2A). None of the three resulting cohesin “hemimutants” showed detectable ATPase activity (Figure 2B), confirming that mutation of Smc3’s ATP binding sites is sufficient to prevent ATP hydrolysis also at Smc1’s NBD [30]. This finding enabled us to analyze the behavior of cohesin ATPase mutants by expressing the above-mentioned Smc3 mutants in HeLa cells and following their behavior in fluorescence recovery after photobleaching (FRAP) experiments, without having to coexpress mutated forms of Smc1 and having to deplete endogenous Smc1 and Smc3. For these experiments, we modified a bacterial artificial chromosome (BAC) containing the mouse *Smc3* locus and a C-terminal localization and affinity purification (LAP) tag by introducing the same point mutations as described above. The Smc3 mutants were stably expressed in HeLa cells at levels close to or below endogenous human Smc3, they assembled with endogenous subunits into cohesin complexes (Figure S2), and the GFP moiety of the LAP tag was used for FRAP analyses.

Previous FRAP experiments had indicated that cohesin diffuses throughout the nucleus and cytosol and binds to unreplicated DNA with a residence time in the range of minutes [31]. This “dynamic binding mode” is thought to be the result of continuous loading of cohesin onto chromatin and subsequent release by Wapl-mediated opening of the exit gate [5, 12, 13, 15, 17]. To measure the chromatin-binding abilities of the ATPase mutants, we simplified the analysis of FRAP experiments by synchronizing cells in G1 phase and by photobleaching only within the nucleus (Figure 2C). To determine the diffusion coefficient of cohesin that is not bound to chromatin, we also analyzed the FRAP redistribution kinetics of cohesin in mitotic cells, where most cohesin is released from chromosomes by the Wapl-dependent “prophase pathway” [16, 17, 31, 32]. We determined a mean (±SEM) diffusion coefficient for WT Smc3-LAP of 2.96 ± 0.19 μm^2^/s (n = 10) after data normalization [33] and integrated this value in the FRAP analysis as a diffusion parameter.

Consistent with previous reports, WT Smc3-LAP in G1 phase did not turn over completely on chromatin within our experimental time frame, indicating that more than 40% of nuclear cohesin was bound to chromatin (Figure 2D). However, in contrast to previous findings [31], our data could not be explained by a single population of cohesin being associated with chromatin (dotted line in Figure 2D). Instead, a sum of two exponential functions (dashed black line in Figure 2D) representing two populations of cohesin with reduced fluorescence recovery was required to describe the data accurately. This indicates that cohesin can interact with unreplicated chromatin in two different modes: one that results in dissociation of cohesin from chromatin within less than 1 min, and another one that results in a residence time of several minutes. The second interaction mode corresponds to the “dynamic binding mode” previously described by Gerlich et al. [31], whereas we refer to the first one as “transient binding mode.” We suspect that the transient binding mode was not detected by Gerlich et al. [31] because in this study, the measurement of fluorescence recovery was only started 1 min after photobleaching.

When we analyzed Smc3 mutants by FRAP, we observed that the Walker A, the signature motif, and the Walker B motif mutant showed faster redistribution kinetics than WT cohesin, indicating that their residence times on DNA were reduced (Figure 2E). However, the fluorescence signals of the ATPase mutants did not recover to the same degree as mitotic WT cohesin (Figure 2D), implying that the ATPase mutants were still able to interact with chromatin. Further analysis of the recovery kinetics indicated that the ATPase mutants interacted with chromatin predominantly through the transient interaction mode, whereas the dynamic binding mode was greatly reduced (Figures 2F and 2G).

These results indicate that cohesin shows two types of chromatin association in G1 phase: (1) a transient association in the range of tens of seconds that does not depend on cohesin’s ATPase activity, and (2) a more long-lasting “dynamic” type of chromatin interaction that depends on cohesin’s ability to hydrolyze ATP.

### The Chromatin Association of Cohesin ATPase Mutants Is Regulated by the Loading Complex, but Not by Wapl

**Figure 3:**
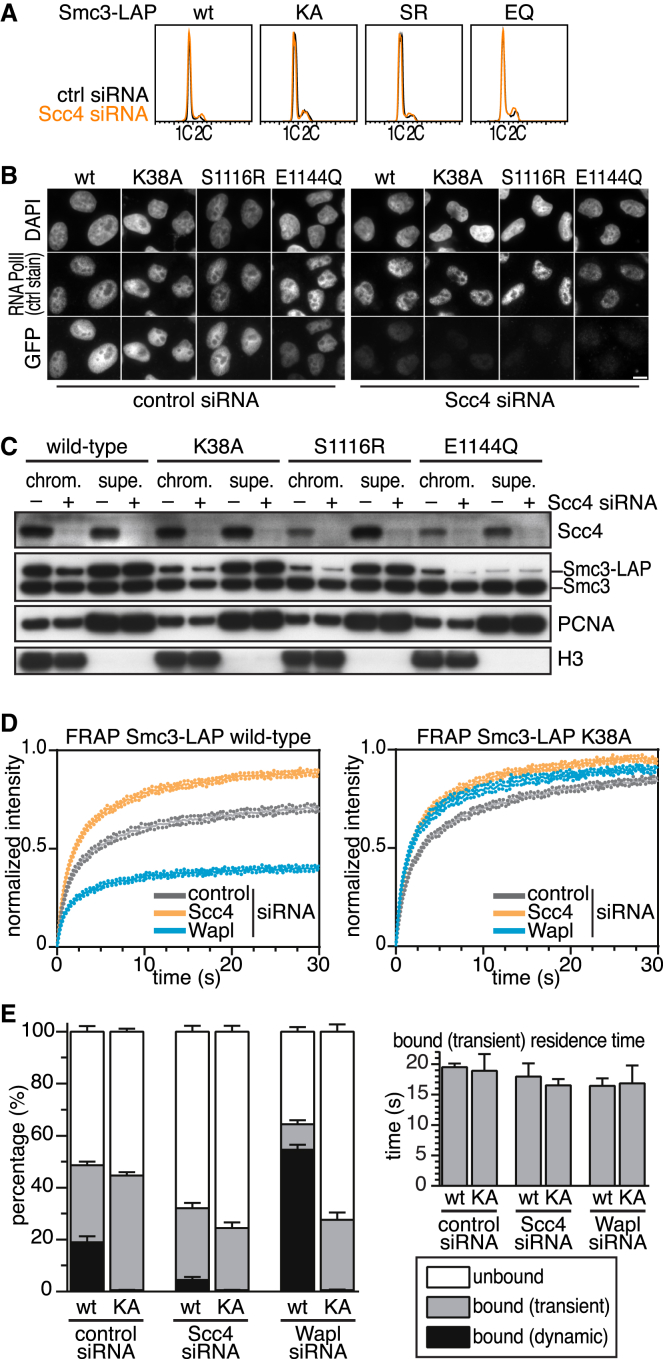
Chromatin Association of Cohesin ATPase Mutants Depends on Scc4, but Not on Wapl (A) Fluorescence-activated cell-sorting profiles of cells synchronized in G1 phase after 48 hr Scc4 depletion and stained with propidium iodide. ctrl, control. (B) Immunofluorescence microscopy experiment showing localization of Smc3-LAP on chromatin after RNAi and pre-extraction before fixation. DAPI was used to stain DNA, RNA polymerase II (PolII) staining was used as a signal intensity control, and GFP antibody was used to detect Smc3-LAP. Scale bar represents 10 μm. (C) Chromatin (chrom.) and soluble (supe.) fractions of cells from (A) and (B) were analyzed by immunoblotting. (D) Quantification of fluorescent signals in FRAP experiments after control, Scc4, or Wapl depletion for 72 hr. (E) Quantification of experiments in (D) to measure unbound, transiently bound, and dynamically bound pools of cohesin and the calculated residence time of the transiently bound state. Error bars in (D) and (E) denote SEM (n ≥ 13 per condition). See also Figure S3.

We next analyzed if the transient chromatin interaction of the ATPase mutants depends on the cohesin loading complex. For this purpose, we synchronized the different HeLa cell lines in G1 phase (Figure 3A) and transfected cells with small interfering RNA (siRNA) specific for Scc4/MAU2 or with control siRNA. Subsequently, we analyzed the levels of Smc3-LAP on chromatin by immunofluorescence microscopy in cells from which soluble proteins had been removed by pre-extraction (Figure 3B), by immunoblotting after separation of cell lysates into chromatin and supernatant fractions (Figure 3C). We also analyzed asynchronously proliferating cells by FRAP (Figures 3D and 3E). Although the FRAP experiments in Figure 2F had indicated that similar proportions of WT and ATPase mutant cohesin are associated with chromatin, we observed in both immunofluorescence microscopy and immunoblotting experiments smaller amounts of the ATPase mutants than WT cohesin on chromatin. We suspect that the reduced levels of the ATPase mutants on chromatin are caused by their short chromatin residence time, which may have led to their partial dissociation from chromatin during sample preparation. Importantly, the low levels of all three ATPase mutants on chromatin were further reduced in cells depleted of Scc4/MAU2 (Figures 3B and 3C), indicating that the association of these mutants with chromatin still depends on the cohesin loading complex. FRAP analysis confirmed this notion because Scc4 depletion increased the recovery time of both WT Smc3 and Smc3 KA by increasing the fraction of freely diffusing cohesin (Figures 3D and 3E). Similar observations have been made in yeast where the chromatin association of an Smc1 ATP hydrolysis mutant (E1158Q) depends on Scc2 [11].

It has been proposed that yeast cohesin is first recruited to DNA by the loading complex and that cohesin’s ATPase activity is subsequently needed to entrap cohesin inside its ring structure [10, 11]. We tested predictions made by this hypothesis by analyzing the behavior of human cohesin ATPase mutants in cells depleted of Wapl. In such cells, WT cohesin accumulates on chromatin [15, 17], presumably because these cohesin complexes entrap DNA inside their ring structure but cannot be released from DNA again because Wapl would be needed to open their exit gate [5, 12, 13]. However, if cohesin ATPase mutants are deficient in the step that entraps DNA inside the cohesin ring, Wapl depletion should not lead to a stabilization of these mutants on chromatin. To test this prediction, we depleted Wapl by RNAi (Figure S3A) in HeLa cells expressing WT or KA mutant Smc3-LAP and analyzed the behavior of these proteins by FRAP (Figures 3D and 3E).

Whereas the recovery of fluorescent WT cohesin was greatly reduced following Wapl depletion, the recovery of ATPase mutant cohesin was not and was instead slightly increased (Figure 3D). Further analysis of the FRAP kinetics revealed that Wapl depletion increased the amount of WT cohesin on chromatin, whereas the amounts of the ATPase mutant cohesin on chromatin were slightly reduced under these conditions (Figures 3E and S3B). These findings are consistent with the hypothesis that cohesin is first recruited to DNA by the cohesin loading complex and subsequently entraps DNA in a step that depends on cohesin’s ATPase activity. We do not know why Wapl depletion slightly increases the recovery of the Smc3-LAP KA mutant in FRAP assays, but it is possible that this is an indirect effect of chromatin compaction that is known to be caused by stabilization of WT cohesin on chromatin in Wapl-depleted cells ([15]; Figure S3B).

### Cohesin Acetylation Does Not Influence Its ATPase Activity

Because Smc3 acetylation occurs in the proximity of cohesin’s ATP binding sites (Figure 1A; [18, 19]) and has been proposed to diminish cohesin’s ATPase activity [25], we attempted to measure the ATPase activity of acetylated cohesin. To generate acetylated cohesin, we first incubated recombinant cohesin complexes with purified human Esco1 and its cofactor, acetyl-coenzyme A (CoA). Although the Esco1 enzyme used in these experiments was able to acetylate cohesin associated with *Xenopus* sperm chromatin [23], we were unable to detect Smc3 acetylation in the purified system (see Figure 5B). We therefore isolated cohesin dimers and trimers from insect cells in which Esco1 had been coexpressed. Under these conditions, Smc3 acetylation could be detected with an acetyl-specific Smc3 antibody [23] in the purified cohesin complexes (Figures S4A, S4B, S4D, and S4E). We estimate that in these samples, approximately half of the cohesin molecules had been acetylated (see legend for Figure S4B). However, the specific ATPase activity of these complexes did not differ significantly from the activity of cohesin samples in which no acetylation could be detected (Figures S4C and S4F).

**Figure 4:**
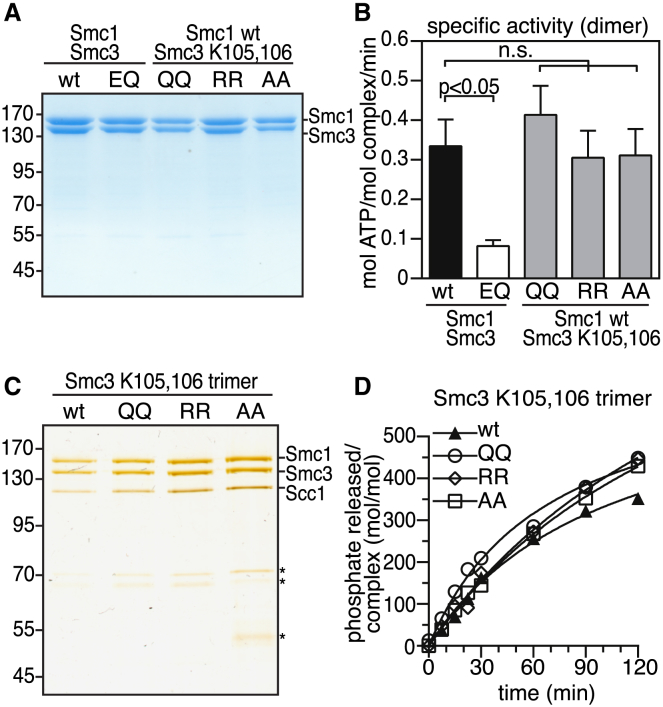
Smc3 Acetylation Does Not Detectably Affect Cohesin’s ATPase Activity (A) Coomassie staining of cohesin dimers mutated at Walker B (Smc1 E1157Q and Smc3 E1144Q) or acetylation sites (Smc3 K105 and K106). (B) Comparison of specific activities of WT and mutant dimeric cohesin complexes in (A). Error bars denote SEM (n ≥ 8). (C) Silver staining of purified cohesin trimers mutated at lysines 105 and 106. Asterisks denote unidentified proteins. (D) Comparison of hydrolysis rates for cohesin complexes from (C). See also Figure S4.

We also generated cohesin complexes containing forms of Smc3 in which K105 and K106 had been mutated to glutamine (QQ), arginine (RR), or alanine (AA; Figures 4A and 4C). These three Smc3 mutants resemble acetylated Smc3 in its ability to bind sororin, which normally only interacts with acetylated but not with nonacetylated cohesin, implying that these mutants functionally mimic the acetylated form of Smc3 [23]. We therefore tested if cohesin dimers and trimers containing the QQ, RR, or AA mutants of Smc3 were altered in their specific ATPase activity. However, this was not the case (Figures 4B and 4D). Taken together, these results indicate that Smc3 acetylation has no major effect on cohesin’s ATPase activity, at least under our assay conditions.

### Cohesin’s ATPase Activity Is Essential for Smc3 Acetylation

**Figure 5:**
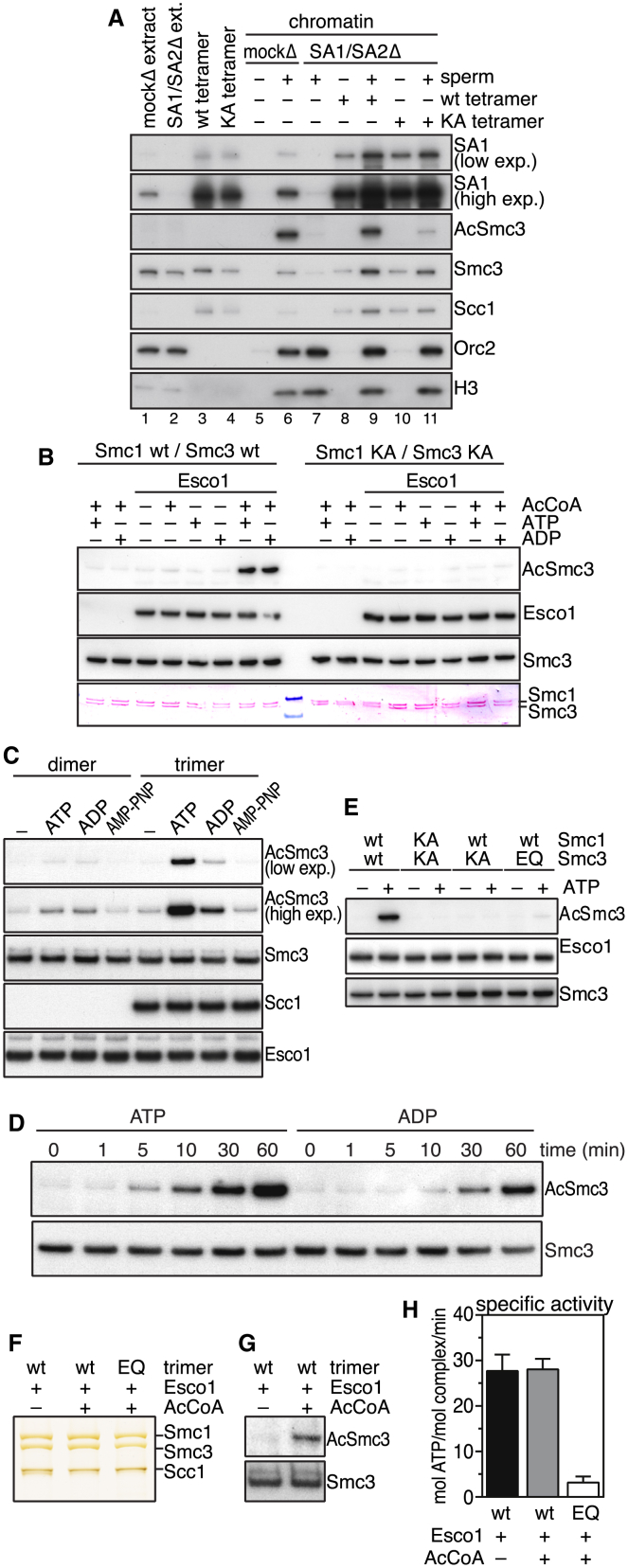
Cohesin’s ATPase Activity Is Required for Smc3 Acetylation (A) *Xenopus* extracts (ext.) were immunodepleted with an antibody against SA1/SA2 (lane 2) and supplemented with human cohesin complexes (lanes 3 and 4) before sperm addition (see also Figures 1H and S1D). Two hours later, chromatin fractions (lanes 6, 7, 9, and 11) and samples without sperm addition (to control for the dependence of cohesin pelleting on chromatin association) were analyzed by immunoblotting. Please note that SA1/SA2 depletion does not lead to complete codepletion of Smc3 (lane 2) because these extracts also contain Smc1-Smc3 heterodimers that are not bound to SA1 or SA2 [34, 35]. However, only a little of the Smc1-Smc3 heterodimer that remains in the extract after SA1/SA2 immunodepletion associates with chromatin (lane 7), and the heterodimer is not expected to contribute to sister chromatid cohesion (see Figure 1H). exp., exposure. (B) Purified dimeric cohesin complexes were incubated with human Esco1, acetyl-CoA, and ATP or ADP (see also Figure S5A). Acetylation and protein levels were analyzed by western blotting and Ponceau staining. (C) Cohesin dimers and trimers were subjected to the acetylation reaction in the presence of ATP, ADP, or AMP-PNP. (D) Trimeric cohesin complexes were subjected to the acetylation reaction in the presence of ATP or ADP, and the degree of acetylation was assayed at the indicated time points. (E) Trimeric cohesin complexes mutated at one or both ATPase domains were assayed in the acetylation reaction in the presence or absence of ATP. (F) Silver staining of purified cohesin trimers (WT or EQ mutant) after incubation with Esco1 and ATP in the presence or absence of acetyl-CoA for 60 min at 37°C. (G) WT cohesin trimers from (F) were analyzed by immunoblotting for the presence of acetylated Smc3. (H) ATP hydrolysis quantification of cohesin trimers in (F) after incubation with 400 μM ATP. Error bars denote SD (n = 3). See also Figure S5.

In the immunoprecipitation experiments shown in Figure S2, we had failed to detect acetylated Smc3 in the cohesin ATPase mutants expressed in HeLa cells, even though we had analyzed similar amounts of ATPase mutant and WT complexes by immunoblotting and had found that WT cohesin was clearly acetylated under these conditions. Furthermore, the ATPase mutant complexes contained little if any sororin, presumably due to the absence of acetylated Smc3 (Figure S2A). Similar results were obtained when WT or Walker A mutant (KA) tetrameric cohesin complexes were added to cohesin-depleted *Xenopus* egg extract and incubated with sperm DNA to initiate cohesin loading and DNA replication. After 120 min, chromatin-bound proteins were analyzed by immunoblotting. Although both WT and KA complexes bound to sperm chromatin, WT cohesin contained much more acetylated Smc3 than the ATPase-deficient complex (Figure 5A, compare lanes 9 and 11).

These results indicate that cohesin ATPase mutants cannot be acetylated. This deficiency could be an indirect consequence of the short residence time of the ATPase mutants on chromatin. Alternatively, cohesin’s ATPase activity could be directly required for the acetylation reaction. To test the latter possibility, we analyzed if purified Esco1 (Figure S5A) could acetylate recombinant cohesin complexes in vitro under conditions where these complexes can hydrolyze ATP. As mentioned above, no Smc3 acetylation could be detected when cohesin dimers were incubated with Esco1 and acetyl-CoA. However, when the same reactions were carried out in the presence of ATP, Smc3 acetylation was readily detectable (Figure 5B). ATP did not have this effect when cohesin Walker A mutants were used as a substrate (Figure 5B), indicating that ATP enabled Smc3 acetylation by affecting cohesin and not Esco1.

When we used cohesin dimers as substrates, ADP promoted Smc3 acetylation similarly well as ATP (Figure 5B), but when we used cohesin trimers, much more Smc3 acetylation was observed in the presence of ATP than of ADP (Figure 5C). Together with the finding that trimers have a higher specific ATPase activity than dimers (Figure 1C), this observation implies that ATP hydrolysis supports Smc3 acetylation more efficiently than ADP binding. Consistent with this interpretation, we found in a time course experiment that ATP enabled the acetylation of cohesin trimers much more rapidly than ADP (Figure 5D). Furthermore, we observed that the nonhydrolyzable ATP analog adenylylimidodiphosphate (AMP-PNP) did not promote Smc3 acetylation above “background” levels (Figure 5C), although it efficiently inhibited ATP hydrolysis by WT cohesin (Figure S5B), suggesting that AMP-PNP bound to these cohesin complexes. Similarly, we observed that trimeric complexes mutated at Smc3’s Walker B motif (EQ) were not acetylated in the presence of ATP (Figure 5E), although these complexes are predicted to bind ATP (but to be unable to hydrolyze it [7]).

To further test if cohesin’s ATPase activity is required for Smc3 acetylation, we also tested if mutation of Smc1’s NBD abrogates Smc3 acetylation (note that all of the above experiments had been carried out with Smc3 hemimutants or Smc1-Smc3 double mutants). As expected, mutation of the Walker A (K38A) and signature (S1129R) motifs in Smc1 reduced Smc3 acetylation of cohesin trimers, but mutation of Smc1’s Walker B motif (E1157Q) had no detectable effect on ATP-dependent Smc3 acetylation (Figures S5C–S5F). However, ATPase assays revealed that the Smc1 EQ hemimutant was still able to hydrolyze ATP at a reduced rate (Figure S5D). This Walker B mutation is thought to prevent ATP hydrolysis but not ATP binding at Smc1’s NBD, raising the possibility that ATP binding at Smc1’s NBD is sufficient to trigger ATP hydrolysis by the Smc3 subunit. Consistent with this interpretation, a point mutation in the Walker B motif of Smc3 (EQ) abrogated the activity of the Smc1 Walker B mutant (Figure S5E). Importantly, this double mutant could not be acetylated (Figure S5B). The ability of Esco1 to acetylate Smc3 therefore correlates with the ability of cohesin to hydrolyze ATP.

Our experiments in which we had coexpressed cohesin and Esco1 in insect cells had indicated that Smc3 acetylation does not significantly alter cohesin’s ATPase activity (see Figure S4). To further test this notion, we also incubated purified cohesin trimers with Esco1 and ATP in either the absence or presence of acetyl-CoA, reisolated cohesin by immunoprecipitation, and measured its ATPase activity (Figure 5F). Also in this experiment, the ATPase activities of both samples were undistinguishable (Figure 5H), despite the fact that Smc3 acetylation occurred in the presence of Esco1, ATP, and acetyl-CoA, but not in the absence of acetyl-CoA (Figure 5G). Also under these conditions, the ATPase activity could be attributed to cohesin because a cohesin trimer in which both Smc1 and Smc3 had been mutated in their Walker B motifs (EQ) showed much less ATP hydrolysis activity (Figure 5H).

## Discussion

Cohesin, first discovered as a protein complex essential for sister chromatid cohesion, is now known to carry out a variety of important functions in both proliferating and postmitotic cells, ranging from DNA repair to chromatin organization and gene regulation. Cohesin mediates all of these functions by interacting with DNA, presumably by topologically entrapping DNA inside its ring structure [4]. Understanding how cohesin entraps DNA and how this interaction is regulated is therefore of great importance.

Previous FRAP experiments had revealed that cohesin can interact with chromatin in two different ways: a dynamic and a stable binding mode [31]. The dynamic binding mode is thought to be the result of continuous cohesin loading and release reactions, mediated by the loading complex and Wapl, respectively, and has been proposed to contribute to chromatin organization and gene regulation [12, 15]. The stable binding mode occurs only during and after DNA replication, depends on Smc3 acetylation and sororin binding, and is thought to be required for mediating cohesion from S phase until mitosis [23, 36]. The FRAP data presented here provide direct evidence for a third, much more transient binding mode with which cohesin can interact with chromatin in the range of seconds. This binding mode might correspond to transient cohesin-chromatin interactions that have previously been observed in *D. melanogaster* [37]. Several observations indicate that this transient interaction represents an intermediate step in the loading reaction. Cohesin ATPase mutants retain the ability to interact with chromatin transiently in a manner that depends on the loading complex, but unlike WT cohesin, these mutants fail to associate with chromatin for longer periods of time in the absence of Wapl. These results provide further support for the hypothesis proposed by Hu et al. [11] and Murayama and Uhlmann [10] that cohesin is initially recruited to DNA via the cohesin loading complex, resulting in a transient cohesin-chromatin interaction, and that subsequent ATP hydrolysis by cohesin is needed to entrap DNA inside the cohesin ring, resulting in the dynamic binding mode. How ATP hydrolysis at cohesin’s ATP binding sites could lead to separation of the hinge domains of Smc1 and Smc3 at the other “end” of cohesin remains a mystery. It has been speculated that ATP hydrolysis might induce a conformational switch that is transmitted via the coiled coils of Smc1 and Smc3 to the hinge regions [6], possibly assisted by multiple contacts of the loading complex with the cohesin ring [10].

The proximity of cohesin’s ATP binding sites to the acetylated lysine residues on Smc3 has led to speculations about possible roles of Smc3 acetylation in controlling cohesin’s ATPase activity [19]. Consistent with such a role, it has been observed that dominant-negative effects of a yeast Smc3 ATPase mutant could be similarly reduced by mutating its acetyl or ATP binding sites [25]. However, in our biochemical assays, we were unable to detect effects of Smc3 acetylation or of mutations introduced into the acetylation sites on cohesin’s ATPase activity. Although we cannot exclude the existence of such effects in the cellular context, we suspect that Smc3 acetylation stabilizes cohesin on chromatin by other mechanisms than inhibiting cohesin’s ATPase activity, namely by recruitment of sororin and inactivation of Wapl.

Unexpectedly, we observed that the opposite was the case: Smc3 acetylation was strictly dependent on cohesin’s ATPase activity in both HeLa cells and *Xenopus* egg extracts. Similar observations have been made for cohesin ATPase mutants in yeast, but in this case, the absence of acetylation has been attributed to the transient association of these mutants with chromatin [38] where Smc3 acetylation occurs [23]. However, our finding that Smc3 acetylation also depends on cohesin’s ATPase activity in a reconstituted system containing recombinant cohesin and Esco1 implies that the short residence time of these mutants on chromatin is not the only and possibly not the main reason why Smc3 does not become acetylated in these mutants. Instead, our results indicate that cohesin’s ability to hydrolyze ATP into ADP is a prerequisite for Smc3 acetylation. Although we found that ADP can also support Smc3 acetylation to some degree, we suspect that under physiological conditions, it is the process of ATP hydrolysis that enables Smc3 acetylation because in cells, the concentration of ATP is much higher than the concentration of ADP, and because ATP supported the acetylation of trimeric cohesin much better and more rapidly than ADP. Because we have so far not been able to generate recombinant active Esco2, we do not know if Smc3 acetylation by this enzyme also depends on cohesin’s ATPase activity. However, we suspect that this is the case because Esco1 and Esco2 modify the same lysine residues on Smc3, and because the acetylation of ATPase mutants was strongly diminished in *Xenopus* egg extracts, which contain little if any Esco1 [22] and in which WT cohesin is therefore exclusively acetylated by Esco2.

**Figure 6:**
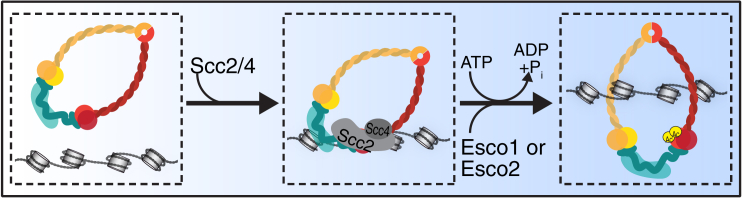
Cohesin’s ATPase Activity Couples Cohesin Loading onto DNA with Smc3 Acetylation Schematic model of cohesin loading and Smc3 acetylation. The cohesin loading complex composed of Scc2/NIPBL and Scc4/MAU-2 mediates association of cohesin with DNA. ATP binding and hydrolysis trigger chromatin entrapment and allow for Smc3 acetylation by Esco1 and presumably Esco2, thereby initiating cohesion establishment.

Why is Smc3 acetylation dependent on cohesin’s ATPase activity? It is plausible to think that ATPase activity converts cohesin into a conformation that makes it susceptible for acetylation. If this conformation can be maintained for longer periods of time, it is conceivable that cohesin loaded onto chromatin in G1 phase would become acetylated later during DNA replication to establish sister chromatid cohesion. Consistent with such a sequential model, it has been proposed that cohesin loaded onto chromatin before DNA replication is sufficient to mediate sister chromatid cohesion later in the cell cycle [29]. Alternatively, it is conceivable that normally, cohesion is established by cohesin complexes that are loaded de novo onto chromatin during DNA replication. Such a scenario could explain in functional terms why Smc3 acetylation depends on cohesin’s ATPase activity. According to this hypothesis, ATP hydrolysis would mediate entrapment of newly synthesized sister DNA molecules and would at the same time convert Smc3 into a state that is susceptible to acetylation (Figure 6). This modification would then, through a poorly understood process, lead to recruitment of sororin and inhibition of Wapl. These events would “lock” the exit gate and thus lead to the stable binding mode of cohesin that is required for sister chromatid cohesion. Although speculative, this hypothesis could explain why in *Xenopus* egg extracts both the loading complex and Esco2 are recruited to prereplicative complexes where DNA replication is initiated [39, 40, 41, 42].

## Author Contributions

V.B., R.L., P.J.H.i.t.V., I.F.D., E.K., G.P., and J.-M.P. designed experiments and interpreted data. V.B. and P.J.H.i.t.V. generated recombinant human cohesin. V.B. performed ATPase and acetylation assays. I.F.D. performed *Xenopus* experiments. E.K. generated Smc3-LAP-expressing cell lines and performed IP-MS experiments. R.L. performed FRAP and RNAi experiments. R.L., V.B., and J.-M.P. wrote the manuscript.

## Supplemental Information

Document S1. Figures S1–S5 and Supplemental Experimental Procedures

Document S2. Article plus Supplemental Information
