# Supplementary material for: Cohesin’s ATPase Activity Couples Cohesin Loading onto DNA with Smc3 Acetylation
Source: Curr Biol. 2014 Oct 6;24(19):2228–37. doi: 10.1016/j.cub.2014.08.011 (PMC4188815; doi:10.1016/j.cub.2014.08.011)
Supplement: Document S1. Figures S1–S5 and Supplemental Experimental Procedures [file mmc1.pdf]

Current Biology, Volume *24*

Supplemental Information

Cohesin's ATPase Activity Couples Cohesin Loading onto DNA with Smc3 Acetylation

Rene Ladurner, Venugopal Bhaskara, Pim J. Huis in 't Veld, Iain F. Davidson, Emanuel Kreidl, Georg Petzold, and Jan-Michael Peters

Supplemental Figures

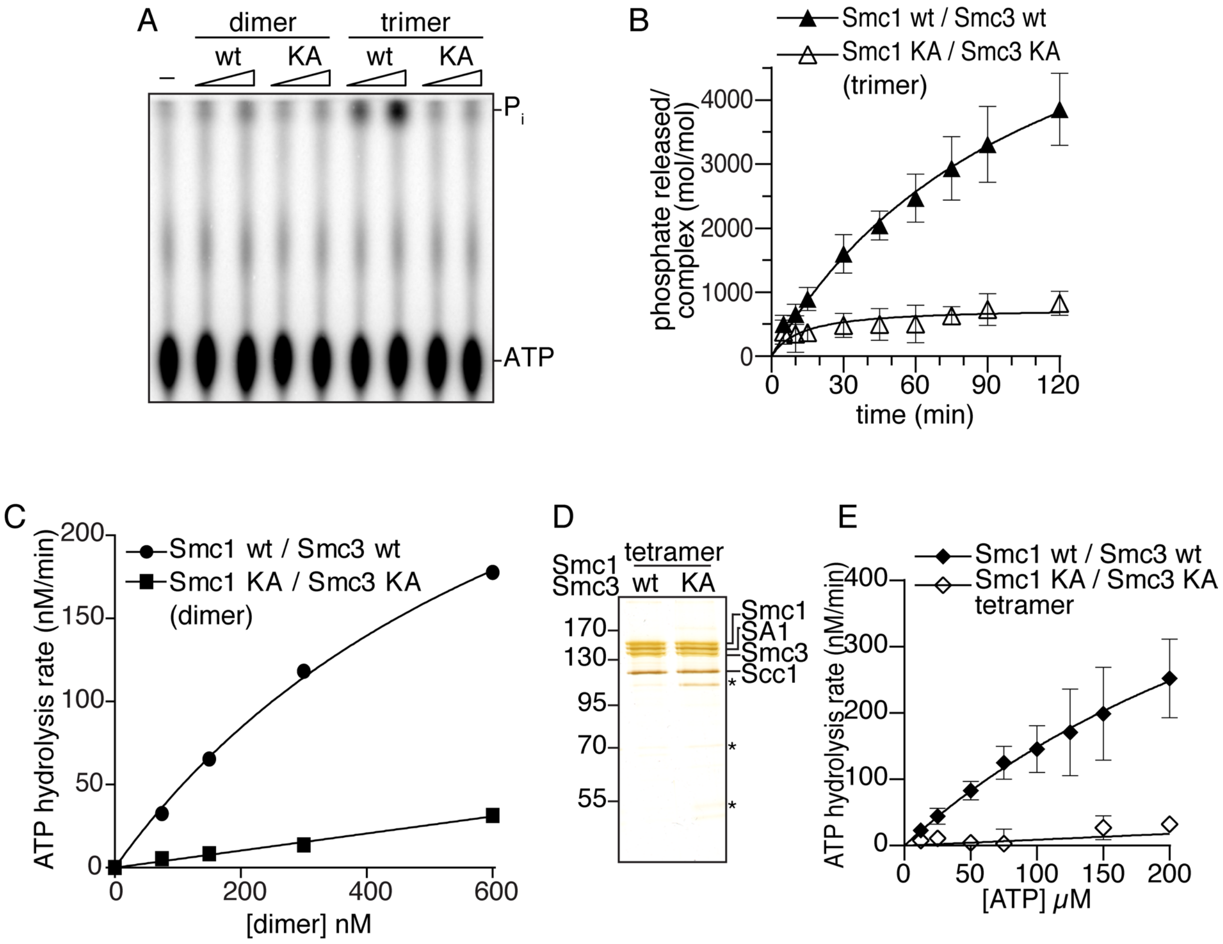

Figure S1, related to Figure 1. Reconstitution and functional characterization of human cohesin

(A) Thin-layer chromatography-autoradiography separated radiolabeled substrates and products of ATP hydrolysis reactions as indicated.
(B) Time course quantification of phosphate released after incubation of purified trimeric cohesin complexes with 400 μM ATP. Error bars denote standard deviation (n = 3).
(C) ATP hydrolysis rate quantification of dimeric cohesin as a function of enzyme concentration.
(D) Purified tetrameric cohesin complexes were analyzed by silver staining. Asterisks denote unidentified proteins.
(E) Substrate dose response quantification of purified complexes to measure ATP hydrolysis rates. Error bars denote standard deviation (n = 2).

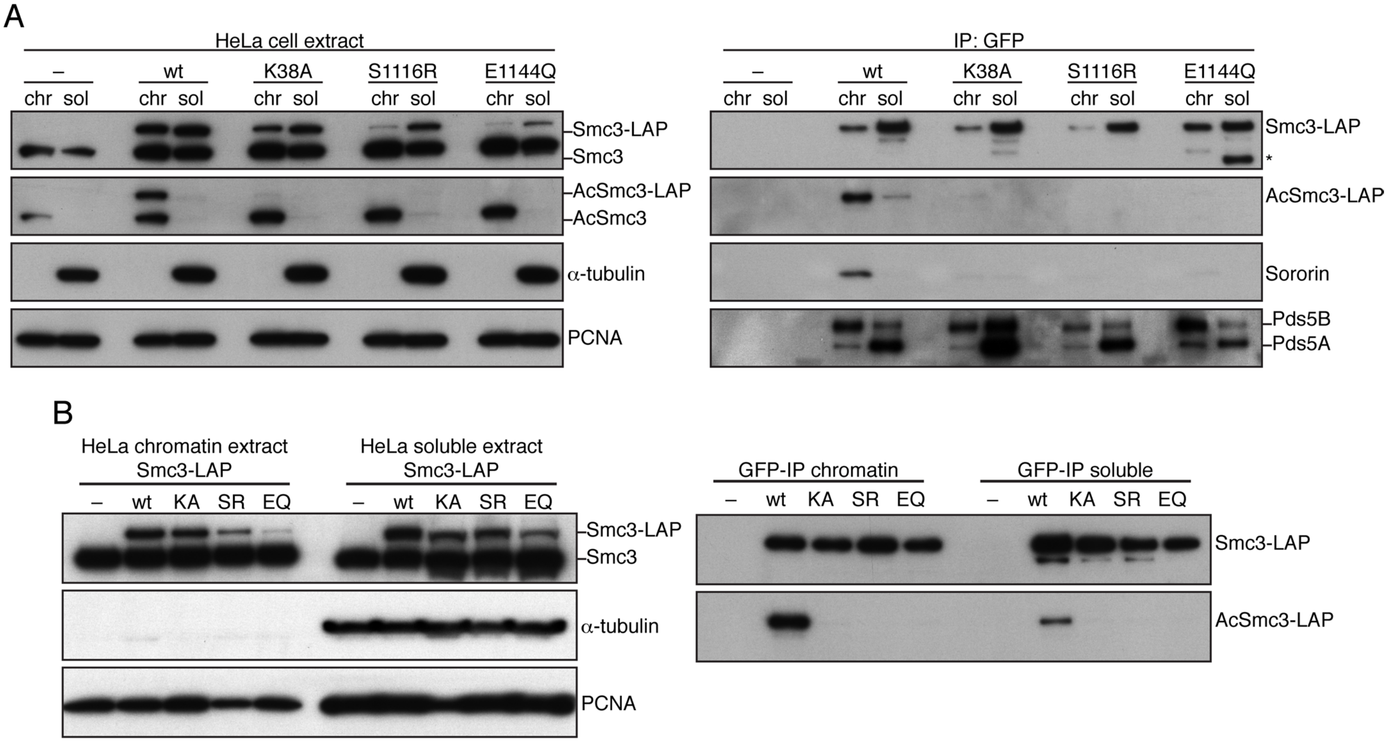

C

		Smc3-LAP			
		wild-type	K38A	S1116R	E1144Q
identified proteins	molecular weight	number of unique peptides identified			
Smc3	142 kDa	117	79	120	111
Smc1A	143 kDa	107	85	124	109
Pds5B	165 kDa	84	52	84	82
SA2	146 kDa	75	43	76	67
Wapal	143 kDa	66	45	65	60
Pds5A	151 kDa	64	22	58	46
SA1	144 kDa	52	52	55	46
Rad21	72 kDa	45	34	46	41
Sororin	28 kDa	14	5	4	4
Esco2	93 kDa	2	0	0	0

Figure S2, related to Figure 2. Cohesin ATPase mutants associate transiently with chromatin

(A) HeLa cells containing Smc3-LAP wild-type and mutants were grown asynchronously and Smc3-LAP was immunoprecipitated from soluble (sol) and nuclease-treated chromatin (chr) fractions as indicated. HeLa cells without Smc3-LAP were used as control. Asterisk denotes cross-reacting protein band.
(B) Cells were synchronized in G2-phase and soluble and nuclease-treated chromatin fractions were prepared and immunoprecipitated as indicated.
(C) Smc3-LAP immunoprecipitation from nuclease-digested chromatin fractions was subjected to MS analysis.

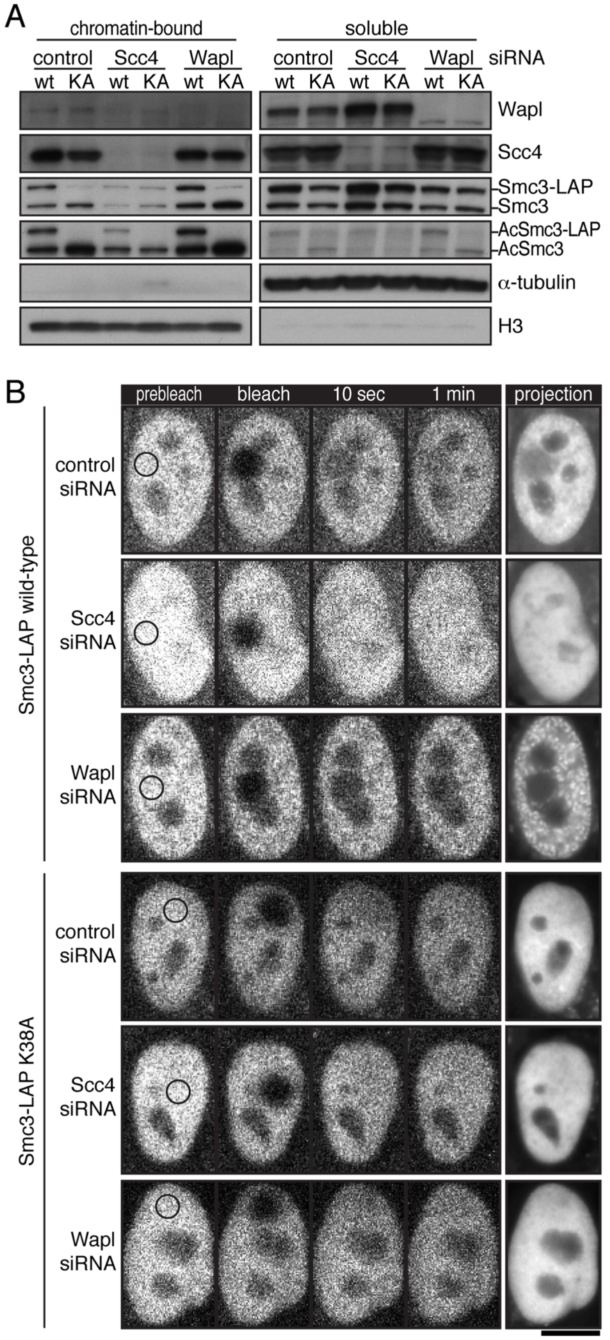

Figure S3, related to Figure 3. Chromatin association of cohesin ATPase mutants depends on Scc4, but not on Wapl

(A) Chromatin and soluble fractions of cells from Figure 3D,E were analyzed by immunoblotting to verify efficient protein depletion. Please note that the chromatin-bound KA mutant extract after Scc4 RNAi contained some α-tubulin, indicating the presence of some soluble material. This might explain why more Smc3-LAP KA is detected on chromatin after Scc4 depletion (lane 4) than after control depletion (lane 2).

(B) Still images of FRAP experiments in Figure 3D,E showing fluorescence recovery at defined time points and image projections of all 520 time frames ('sum slices'). Note that wild-type Smc3-LAP but not Smc3-LAP KA is seen in speckled structures after depletion of Wapl. These structures might represent chromatin regions in which cohesin is stably bound, similar to how cohesin is seen in axial structures ('vermicelli') in *Wapl*$^{-/\Delta}$ mouse embryonic fibroblasts [S1]. The observation that Smc3-LAP KA is not enriched in such speckles is consistent with the notion that this mutant cannot be stabilized on chromatin by Wapl depletion. Scale bar represents 10 μm.

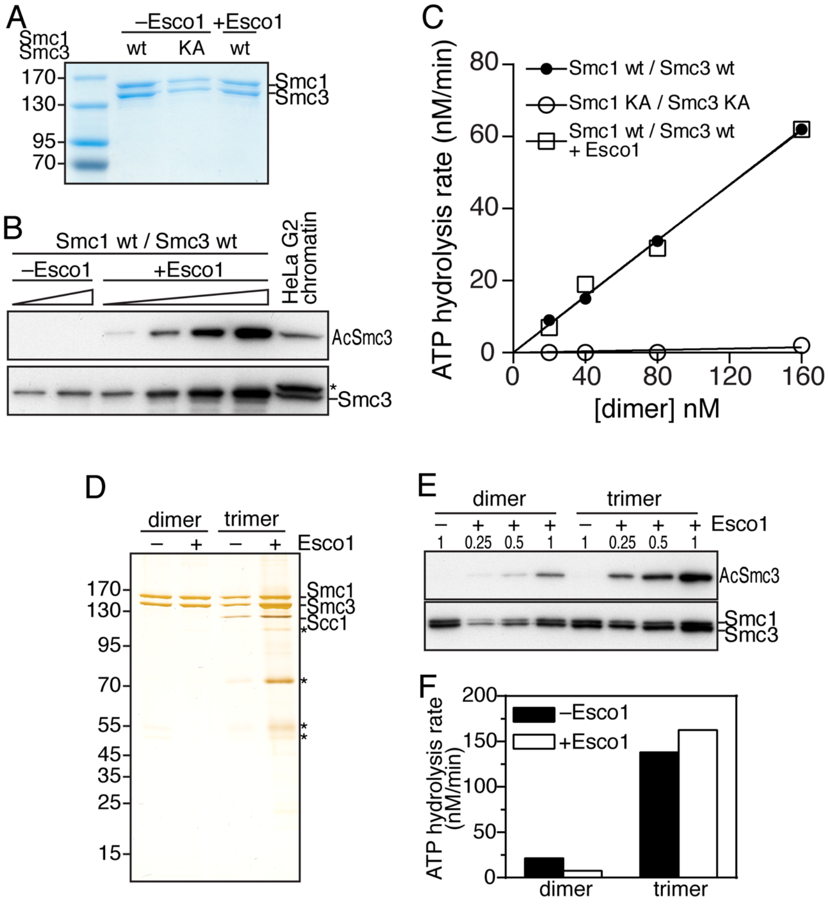

Figure S4, related to Figure 4. Smc3 acetylation does not detectably affect cohesin's ATPase activity

(A) Coomassie staining of cohesin dimers expressed in the presence or absence of human Esco1 and purified from insect cells.
(B) Cohesin complexes from (A) were immunoblotted for Smc3 and acetylated Smc3. Increasing amounts of purified proteins were loaded and compared to acetylation levels of fractionated chromatin from HeLa cells synchronized in G2-phase. Asterisk denotes cross-reacting protein band. Note that co-expression of cohesin dimers with Esco1 results in levels of Smc3 acetylation that are comparable to the levels of Smc3 acetylation that are observed in chromatin fractions of HeLa cells synchronized in G2-phase. Because in G2-phase about half of all chromatin bound cohesin complexes are stably bound [S2] and these are thought to be stabilized by sororin [S3] which only binds to acetylated cohesin [S4, S5], these results imply that in the sample containing recombinant cohesin dimer approximately half of the molecules were acetylated.
(C) Quantification of ATP hydrolysis rate from samples in (A) as a function of enzyme concentration.
(D) Silver staining of cohesin complexes expressed with or without Esco1. Asterisks denote unidentified proteins.
(E) Cohesin complexes from (D) were immunoblotted for Smc1, Smc3 and acetylated Smc3. Increasing amounts of acetylated extracts were loaded to compare acetylation levels between dimeric and trimeric complexes.
(F) Quantification of ATP hydrolysis of cohesin expressed in the presence or absence of Esco1.

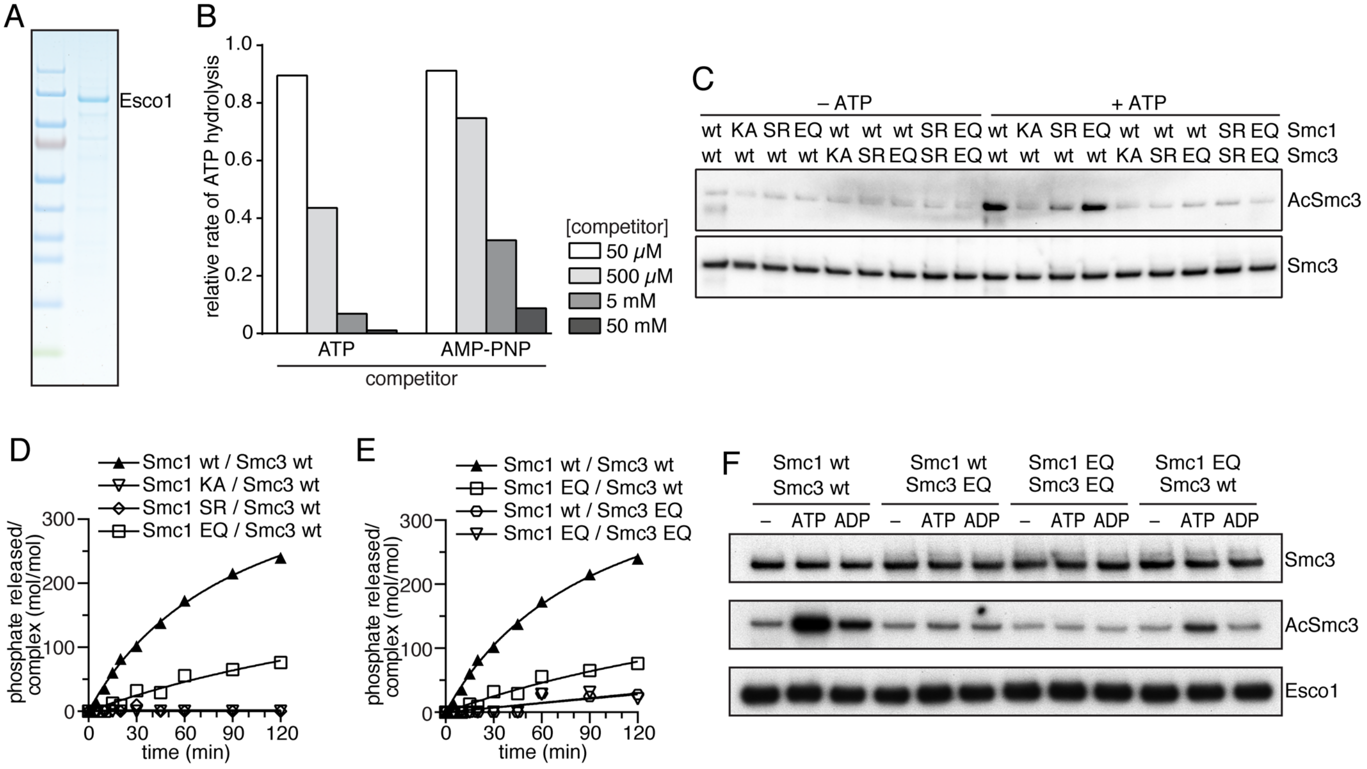

Figure S5, related to Figure 5. Cohesin's nucleotide-bound state is important for Smc3 acetylation.

(A) Purified human Esco1 was analyzed by Coomassie staining.
(B) Quantification of relative ATP hydrolysis rates after supplementing a reaction containing 50 μM ATP and 12.5 nM γ-ATP with different concentrations of competitor.
(C) Different mutant cohesin trimers were subjected to the acetylation reaction in the absence or presence of ATP.
(D) Time course quantification of phosphate released from ATPase reactions with trimeric cohesin containing Smc1 mutations.
(E) Time course quantification of phosphate released from ATPase reactions with Walker B mutation-containing cohesin trimers.
(F) Wild-type and Walker B mutant complexes were subjected to the acetylation reaction in the presence of ATP or ADP. Note that Smc3 acetylation was not increased by ADP if the Walker B motif of Smc1 and/or Smc3 had been mutated (Smc1 EQ, Smc3 EQ), even though these mutants are predicted to be able to bind ADP. This implies either that ADP binding is not sufficient for Smc3 acetylation, or that these mutants cannot bind ADP as well as is predicted.

Supplemental Experimental Procedures

Antibodies. Antibodies against the following proteins were described previously: SA1 (A823); SA1/SA2 (A786); Smc3 (A845, human; A846, *Xenopus*) [S6]; Scc4 (A974, [S7]); Wapl (A961, [S8]); Sororin (A953, [S3]); Esco1 (A782); Scc1 (A900); acetyl-Smc3 (A683, [S5]). Polyclonal rabbit anti-*Xenopus* XCAP-E and Orc2 antisera were kindly provided by E. Watrin and J.J. Blow respectively [S9, S10]. Goat anti-GFP antibody for immunoprecipitation was a kind gift from A.A. Hyman.

The following commercial antibodies were used: Anti-GFP antibody (Abcam, ab13970); Smc1 antibody (Bethyl laboratories, A300-55A); PCNA antibody (Santa Cruz, sc-56); histone H3 antibody (Cell Signaling, 9715L or Santa Cruz, sc-8654); Scc2 antibody (Absea, 010702F01 and 010516H10).

Plasmids and Proteins. Human Smc1-His$_6$, Smc3-FLAG and HA-Scc1 were cloned into pFastbac1 (Invitrogen) and Esco1 was cloned in frame with the His$_6$ in pFastbac-HTC (Invitrogen). Point mutations in Smc1 and Smc3 were introduced by PCR. Bacmids and Baculoviruses were generated and used for protein expression in Sf9 insect cells according to Invitrogen's bac-to-bac manual.

Dimers were expressed by coinfection of Smc1-His$_6$ and Smc3-FLAG viruses. In order to express tetrameric complexes, Smc1, Smc3-FLAG, Scc1 and His$_{10}$-SA1 were combined on a pFL multibac vector [S11]. Trimeric cohesin complexes used in Figure 1 were expressed by combining Smc1, Smc3-FLAG, Scc1-His$_{10}$ on a pFL multibac vector whereas trimers used in subsequent figures were expressed in Sf9 insect cells by co-infection. Human Esco1 was cloned in frame with the His$_6$ tag in pFastbac-HTC for protein expression.

Frozen cell pellets were dounce-homogenized in 2.5 pellet volumes of buffer A (25 mM Tris pH 8.0, 100 mM NaCl, 10% glycerol) supplemented with 5 mM PMSF and 0.5% Tween-20. Lysates were clarified by centrifugation using a 70Ti rotor for 1 hour at 45,000 rpm and incubated with anti-FLAG M2 beads (Sigma) or with 1 mg ml^{-1} anti-HA (12CA5) bound to Bio-Rad Protein A agarose beads for 2 hours to purify dimers and trimers respectively. Bound proteins were washed three times with 10 bead volumes of buffer A containing 0.05% Tween-20 and eluted three times using one bead volume FLAG or HA peptide (0.1 mg ml^{-1}). The eluates were supplemented with 15 mM imidazole and incubated with Ni-NTA beads (Qiagen). Bound proteins were washed with buffer B (150 mM NaCl, 25 mM sodium phosphate pH 7.5, 10% glycerol, 20 mM β-mercaptoethanol, 15 mM imidazole) and eluted with buffer B containing 500 mM imidazole. Proteins were dialyzed against buffer A (slide-a-lyzer dialysis cassette; Pierce 66110), aliquoted and snap-frozen.

Esco1 was expressed in Sf9 cells, purified using buffer B supplemented with 0.5% Tween-20 and 5 mM PMSF and processed as described above.

Sea urchin Δ90 cyclin B was prepared as described [S12].

ATPase assays. Cohesin complexes were incubated in 10 *μ*l reactions containing buffer A with 0.1 mg ml^{-1} BSA, 1 mM $MgCl_2$, 10 nM [γ-^{32}P] ATP and 50 *μ*M cold ATP unless stated otherwise. Reactions were incubated at 37°C and stopped by adding 1% SDS and 10 mM EDTA at the indicated time points. One *μ*l of the reaction was spotted on a polyethyleneimide plate (EMD Biosciences) and the reaction products were separated by TLC using 0.75 M KH_2PO_4 (pH 3.4). The plates were dried and analyzed using a phosphorimager (Bio-Rad). The relative levels of P_i were used as a measure of the ATPase activity.

Acetylation Assays. Acetylation assays were performed in 10 μl reactions containing buffer A supplemented with 0.05 mg ml^{-1} BSA, 1 mM $MgCl_2$, 10 μM acetyl-CoA, 50 nM Esco1 and 150 nM cohesin complex. Reactions were performed in the absence or presence of 50 μM ATP, ADP or AMP-PNP at 37 °C for 1 hr and stopped by adding Laemmli buffer. Antibody A683 after immunoblotting was used to detect the acetylation of Smc3.

Animal experimentation. Experiments on living animals were approved by the IMP IMBA Institutional Animal Welfare Board in accordance with the Austrian Animal Experiments Act of 2012 and Directive 2010/63/EU.

***Xenopus* Egg Extract Preparation and Immunodepletion.** *Xenopus* egg extracts were prepared as described [S13]. Cycloheximide (250 μg ml^{-1}), 25 mM phosphocreatine, 15 μg ml^{-1} creatine phosphokinase, and 0.3 mM $CaCl_2$ were added to extract before use.

Demembranated *Xenopus* sperm nuclei were prepared as described previously [S14]. Sperm was added at 3333 nuclei per μl extract for chromatin isolation experiments and 1111 nuclei per μl extract for immunofluorescence microscopy of *Xenopus* chromosomes. Following sperm addition, extracts were incubated at 23 °C for 2 hours to allow completion of DNA replication and then either driven into mitosis or processed for chromatin isolation. To induce mitosis, nondegradable Δ90 cyclin B was added to egg extract at 300 nM and incubated for a further 3 hours prior to mitotic chromosome isolation and immunofluorescence analysis.

For immunodepletion of cohesin from *Xenopus* egg extracts, two rounds of immunodepletion were performed using an anti-SA1 antibody (A823) followed by a single round using an anti-SA1/SA2 antibody (A786). Affinity-purified antibodies were conjugated to Affi-Prep Protein A Matrix (Bio-Rad) at a ratio of 8.3 μg antibody per μl of beads. Beads were added to interphase

extracts (15 μl beads per 100 μl extract) and incubated on a rotator for 40 min at 4 ^{o}C. Beads were removed by centrifugation.

Preparation of *Xenopus* Chromatin Fractions. Extract was diluted 25-fold in ice cold nuclear isolation buffer (50 mM HEPES-KOH pH 7.6, 50 mM KCl, 5 mM $MgCl_2$, 2 mM DTT, 0.5 mM spermidine, 0.15 mM spermine and 1:1000 protease inhibitor mix) supplemented with 0.1% Triton X-100 and underlayed with 100 μl of this buffer plus 15% sucrose. Samples were centrifuged in a swinging bucket rotor (2100 g, 5 min, 4 ^{o}C) and the pellet recentrifuged in a fixed-angle rotor (15000 g, 2 min, 4 ^{o}C) and resuspended in SDS sample buffer.

Immunofluorescence Microscopy of *Xenopus* Chromosomes. For XCAP-E staining of mitotic *Xenopus* chromosomes, extracts were fixed for 15 min at room temperature with 50 volumes of XBE2 (10 mM HEPES pH 7.7, 100 mM KCl, 2 mM $MgCl_2$, 0.1 mM $CaCl_2$, 5 mM EGTA, 50 mM sucrose) supplemented with 2% formaldehyde and 0.25% Triton X-100. The specimens were layered on top of 30% glycerol in XBE2 and spun onto poly-Lysine-coated coverslips at 3000 g for 15 min. Coverslips were then incubated with 0.5% Triton X-100 in PBS for 5 min, blocked with 3% BSA in PBS, and incubated with anti-XCAP-E antibody.

BAC mutagenesis, HeLa cell culture, RNA interference and FACS. Mouse Smc3-LAP bacterial artificial chromosome mutants were generated as described previously [S5] and transfected into HeLa Kyoto cells. siRNA duplexes were pre-mixed with RNAiMax (Invitrogen) according to manufacturer's instructions, added directly to cells at final concentrations of 30 nM and incubated for 48 or 72 hours. For G1-phase synchronization, cells were treated with 2 mM thymidine for 24 hours and released into fresh medium for 15 hours. Cells for FRAP experiments were grown in 8-well Labtek II chambered coverglass (Nunc). Fifteen minutes

prior to imaging, Hoechst was added at a final concentration of 0.2 µg ml^{-1} to visualize DNA and cycloheximide was used at 1 µg ml^{-1} to inhibit protein synthesis.

Synthetic siRNA oligonucleotides were purchased from Ambion. Sense sequences of siRNA oligos are: Scc4 GAAUUGUACUGUCAAGAGAtt; Wapl CGGACUACCCUUAGCACAAtt. siRNA against firefly GL2 luciferase was used as a control [S15].

Cell-cycle profiling was performed using propidium iodide staining as previously described [S16] with minor modifications. In brief, cells were resuspended in 0.8 ml PBS and fixed with 2.2 ml ice-cold methanol. Cells were washed with PBS, resuspended in PI buffer (50 μg ml^{-1} propidium iodide, 10 mM Tris pH 7.5, 5 mM $MgCl_2$, 200 μg ml^{-1} RNase A) and analyzed using a FACS Canto flow cytometer and FACSDiva software (BD Biosciences).

HeLa cell extracts, immunoblotting and immunoprecipitation. Cell pellets were resuspended in extraction buffer (25 mM Tris pH 7.5, 100 mM NaCl, 5 mM $MgCl_2$, 0.2% NP-40, 10% glycerol, 10 mM sodium butyrate, ‘complete’ protease inhibitor mix (Roche)) and lysed on ice by passing through a hypodermic needle. To fractionate soluble and chromatin-bound proteins, the homogenate was spun at 1300 *g* and washed three times with extraction buffer. Pellets were resuspended in Laemmli’s sample buffer, heated to 95 °C, and passed over a 0.45 μm filter. Immunoblotting was performed as described [S7]. To release proteins from chromatin, samples were treated with benzonase (250 U ml^{-1}) and insoluble material was removed by centrifugation. Supernatant extract was concentration adjusted, added to crosslinked antibody beads, incubated, washed, and eluted with 0.1 M glycine pH 2.0. To analyze interacting proteins by mass spectrometry as described in Figure S2, cells were synchronized in G2-phase by a 6 h release from thymidine arrest and chromatin extracts were

prepared as described above with a single washing step. Immunoprecipitation was performed using crosslinked anti-GFP antibody beads, and eluates were processed for in-solution digest and mass spectrometry as described [S17].

HeLa cell immunofluorescence microscopy. Cells grown on coverslips were pre-extracted with 0.1% Triton X-100 for 2 min as described previously [S18], PBS-washed and fixed with 4% paraformaldehyde in PBS. After fixation, cells were permeabilized with 0.1% Triton X-100 in PBS for 5 min, blocked with 3% BSA in PBS containing 0.01% Triton X-100 and incubated with primary and secondary antibodies (Alexa Fluor 488, Alexa Fluor 568 and Alexa Fluor 647; Molecular Probes). DNA was counterstained with DAPI. Coverslips were mounted onto slides with ProLong Gold (Molecular Probes).

Images were taken on a Zeiss Axioplan 2 microscope with 63x Plan-Apochromat objective lense (Zeiss). The system was equipped with a CoolSnapHQ CCD camera (Photometrics).

Fluorescence recovery after photobleaching (FRAP). Cells synchronized in G1-phase were imaged on an LSM5 Duo (Zeiss) confocal microscope using a 63x Plan-Apochromat objective and open pinhole. Twenty images were acquired before bleaching a radial spot (r = 2 µm) three times at 100% laser intensity (100 mW diode 488). Five hundred images were acquired afterwards at 200 ms intervals. Signal intensities were measured using ImageJ at bleached, nuclear and background regions and normalized according to Ellenberg *et al.* [S19]. Data was analyzed using Berkeley Madonna and a sum of three exponential functions that represent nuclear diffusing, transiently chromatin associated and chromatin bound populations a=ini*((1-(perctrans+percstabl))*(1-EXP(-(kOffdiff)*time))+perctrans*(1-EXP(-(kOfftrans)*time))

+percstabl*(1-EXP(-(kOffstabl)*time))). The diffusion parameter D was measured using the formula $I(t) = I_{final} * (1 - sqrt(4r^2/(4r^2+4*\pi*D*t)))$.

Data quantification and analysis. Quantifications were processed with Microsoft Excel 2007 and GraphPad Prism 6.

Supplemental References

S1. Tedeschi, A., Wutz, G., Huet, S., Jaritz, M., Wuensche, A., Schirghuber, E., Davidson, I.F., Tang, W., Cisneros, D.A., Bhaskara, V., *et al.* (2013). Wapl is an essential regulator of chromatin structure and chromosome segregation. Nature *501*, 564-568.

S2. Gerlich, D., Koch, B., Dupeux, F., Peters, J.M., and Ellenberg, J. (2006). Live-cell imaging reveals a stable cohesin-chromatin interaction after but not before DNA replication. Curr. Biol. *16*, 1571-1578.

S3. Schmitz, J., Watrin, E., Lenart, P., Mechtler, K., and Peters, J.M. (2007). Sororin is required for stable binding of cohesin to chromatin and for sister chromatid cohesion in interphase. Curr. Biol. *17*, 630-636.

S4. Lafont, A.L., Song, J., and Rankin, S. (2010). Sororin cooperates with the acetyltransferase Eco2 to ensure DNA replication-dependent sister chromatid cohesion. Proc. Natl. Acad. Sci. U. S. A. *107*, 20364-20369.

S5. Nishiyama, T., Ladurner, R., Schmitz, J., Kreidl, E., Schleiffer, A., Bhaskara, V., Bando, M., Shirahige, K., Hyman, A.A., Mechtler, K., *et al.* (2010). Sororin mediates sister chromatid cohesion by antagonizing Wapl. Cell *143*, 737-749.

S6. Sumara, I., Vorlaufer, E., Gieffers, C., Peters, B.H., and Peters, J.M. (2000). Characterization of vertebrate cohesin complexes and their regulation in prophase. J. Cell Biol. *151*, 749-762.

S7. Watrin, E., Schleiffer, A., Tanaka, K., Eisenhaber, F., Nasmyth, K., and Peters, J.M. (2006). Human Scc4 is required for cohesin binding to chromatin, sister-chromatid cohesion, and mitotic progression. Curr. Biol. *16*, 863-874.

S8. Kueng, S., Hegemann, B., Peters, B.H., Lipp, J.J., Schleiffer, A., Mechtler, K., and Peters, J.M. (2006). Wapl controls the dynamic association of cohesin with chromatin. Cell *127*, 955-967.

S9. Oehlmann, M., Score, A.J., and Blow, J.J. (2004). The role of Cdc6 in ensuring complete genome licensing and S phase checkpoint activation. J. Cell Biol. *165*, 181-190.

S10. Watrin, E., Cubizolles, F., Osborne, H.B., Le Guellec, K., and Legagneux, V. (2003). Expression and functional dynamics of the XCAP-D2 condensin subunit in Xenopus laevis oocytes. J. Biol. Chem. *278*, 25708-25715.

S11. Fitzgerald, D.J., Berger, P., Schaffitzel, C., Yamada, K., Richmond, T.J., and Berger, I. (2006). Protein complex expression by using multigene baculoviral vectors. Nat Methods *3*, 1021-1032.

S12. Glotzer, M., Murray, A.W., and Kirschner, M.W. (1991). Cyclin is degraded by the ubiquitin pathway. Nature *349*, 132-138.

S13. Gillespie, P.J., Gambus, A., and Blow, J.J. (2012). Preparation and use of Xenopus egg extracts to study DNA replication and chromatin associated proteins. Methods *57*, 203-213.

S14. Lebofsky, R., Takahashi, T., and Walter, J.C. (2009). DNA replication in nucleus-free Xenopus egg extracts. Methods Mol. Biol. *521*, 229-252.

S15. Elbashir, S.M., Harborth, J., Lendeckel, W., Yalcin, A., Weber, K., and Tuschl, T. (2001). Duplexes of 21-nucleotide RNAs mediate RNA interference in cultured mammalian cells. Nature *411*, 494-498.

S16. Wendt, K.S., Yoshida, K., Itoh, T., Bando, M., Koch, B., Schirghuber, E., Tsutsumi, S., Nagae, G., Ishihara, K., Mishiro, T., *et al.* (2008). Cohesin mediates transcriptional insulation by CCCTC-binding factor. Nature *451*, 796-801.

S17. Gregan, J., Riedel, C.G., Petronczki, M., Cipak, L., Rumpf, C., Poser, I., Buchholz, F., Mechtler, K., and Nasmyth, K. (2007). Tandem affinity purification of functional TAP-tagged proteins from human cells. Nat. Protoc. *2*, 1145-1151.

S18. Hauf, S., Roitinger, E., Koch, B., Dittrich, C.M., Mechtler, K., and Peters, J.M. (2005). Dissociation of cohesin from chromosome arms and loss of arm cohesion during early mitosis depends on phosphorylation of SA2. PLoS Biol. *3*, e69.

S19. Ellenberg, J., Siggia, E.D., Moreira, J.E., Smith, C.L., Presley, J.F., Worman, H.J., and Lippincott-Schwartz, J. (1997). Nuclear membrane dynamics and reassembly in living cells: targeting of an inner nuclear membrane protein in interphase and mitosis. J. Cell Biol. *138*, 1193-1206.
