# Supplementary material for: Cohesin’s ATPase Activity Couples Cohesin Loading onto DNA with Smc3 Acetylation
Source: Curr Biol. 2014 Oct 6;24(19):2228–37. doi: 10.1016/j.cub.2014.08.011 (PMC4188815; doi:10.1016/j.cub.2014.08.011)
Supplement: Document S2. Article plus Supplemental Information [file mmc2.pdf]

Current Biology 24, 2228–2237, October 6, 2014 ©2014 The Authors http://dx.doi.org/10.1016/j.cub.2014.08.011

Article

Cohesin's ATPase Activity Couples Cohesin Loading onto DNA with Smc3 Acetylation

Rene Ladurner,[1,2] Venugopal Bhaskara,[1,2,3]
Pim J. Huis in 't Veld,[1] Iain F. Davidson,[1] Emanuel Kreidl,[1]
Georg Petzold,[1] and Jan-Michael Peters[1,*]
[1]Research Institute of Molecular Pathology, Dr. Bohr-Gasse 7, 1030 Vienna, Austria

Summary

Background: Cohesin mediates sister chromatid cohesion by topologically entrapping sister DNA molecules inside its ring structure. Cohesin is loaded onto DNA by the Scc2/NIPBL-Scc4/MAU2-loading complex in a manner that depends on the adenosine triphosphatase (ATPase) activity of cohesin's Smc1 and Smc3 subunits. Subsequent cohesion establishment during DNA replication depends on Smc3 acetylation by Esco1 and Esco2 and on recruitment of sororin, which "locks" cohesin on DNA by inactivating the cohesin release factor Wapl.

Results: Human cohesin ATPase mutants associate transiently with DNA in a manner that depends on the loading complex but cannot be stabilized on chromatin by depletion of Wapl. These mutants cannot be acetylated, fail to interact with sororin, and do not mediate cohesion. The absence of Smc3 acetylation in the ATPase mutants is not a consequence of their transient association with DNA but is directly caused by their inability to hydrolyze ATP because acetylation of wild-type cohesin also depends on ATP hydrolysis.

Conclusions: Our data indicate that cohesion establishment involves the following steps. First, cohesin transiently associates with DNA in a manner that depends on the loading complex. Subsequently, ATP hydrolysis by cohesin leads to entrapment of DNA and converts Smc3 into a state that can be acetylated. Finally, Smc3 acetylation leads to recruitment of sororin, inhibition of Wapl, and stabilization of cohesin on DNA. Our finding that cohesin's ATPase activity is required for both cohesin loading and Smc3 acetylation raises the possibility that cohesion establishment is directly coupled to the reaction in which cohesin entraps DNA.

Introduction

During DNA replication, newly synthesized DNA molecules become physically connected with each other. This sister chromatid cohesion enables the biorientation of chromosomes on the mitotic spindle and is therefore essential for proper chromosome segregation [1]. Cohesion is mediated by the ring-shaped cohesin complex (reviewed in [2]), which contains a heterodimer of the highly elongated Smc1 and Smc3 proteins. Both of these contain long intramolecular coiled coils, a "hinge" region at their central folds, and a nucleotide-binding domain (NBD), which is jointly formed by their N and C termini (Figure 1A). Smc1 and Smc3 dimerize via their hinge domains, whereas association of their NBDs results in the formation of an ABC transporter-like adenosine triphosphatase (ATPase) domain that can bind and hydrolyze two ATP molecules. The "kleisin" subunit Scc1 (also known as Rad21 or Mcd1) bridges the NBDs of Smc1 and Smc3, resulting in a tripartite ring structure. Scc1 is associated with a fourth subunit, called Scc3 in yeast, which in somatic vertebrate cells exists in two isoforms: SA1 and SA2.

Cohesin mediates cohesion by entrapping sister chromatids inside its ring structure [4]. Chromatin fibers have been proposed to enter the cohesin ring via an "entry gate" that is thought to be located between the hinge regions of Smc1 and Smc3 [5, 6]. The loading of cohesin onto chromatin requires cohesin's ATPase activity [7, 8] and a separate loading complex, consisting of the proteins Scc2/NIPBL and Scc4/MAU2 [9, 10]. Experiments in yeast have shown that cohesin complexes deficient in ATP hydrolysis associate with chromatin in a Scc2-dependent but transient manner [11], whereas topological loading of cohesin onto DNA in vitro is stimulated by the loading complex and depends on cohesin's ATPase activity [10]. These observations suggest that the loading complex targets cohesin to chromatin, whereas the ATPase reaction mediates entrapment of DNA inside the cohesin ring.

Once properly loaded, cohesin can be released from DNA by disengagement of the Smc3-Scc1 interface [5, 12, 13]. The opening of this "exit gate" is under the control of Scc3 and the cohesin-interacting proteins Pds5 and Wapl [12, 14]. Cohesin release via the exit gate is thought to contribute to dynamic noncanonical functions of the complex, such as regulation of chromatin structure and gene expression (discussed in [12, 15]), and in vertebrates is used to remove cohesin from chromosome arms in early mitosis [5, 16, 17].

To be able to mediate sister chromatid cohesion, cohesin's exit gate has to be "locked" to prevent precocious release of cohesin from chromatin. To achieve this, the Smc3 subunit is acetylated during DNA replication on two conserved Lys residues (K105,106 in vertebrates) by Eco1 proteins [18–20]. In vertebrates, two Eco1 orthologs exist: Esco2, which is expressed during DNA replication, and Esco1, which is present throughout the cell cycle [21]. Smc3 acetylation during DNA replication leads to the association of cohesin with sororin [22, 23], a protein that inhibits Wapl and thereby prevents opening of the exit gate [5, 12, 13, 23]. Sororin is essential for cohesion in vertebrates and *D. melanogaster* [23, 24], but no sororin ortholog has yet been identified in fungi. In these organisms, cohesin acetylation has been proposed to prevent cohesin release by modulating cohesin's ATPase activity [25] or through interactions between Smc3 and Wapl [26].

Although cohesion can only be established during S phase [27], cohesin is loaded onto DNA already before DNA replication [28], presumably by entrapping a single chromatid fiber. It has been proposed that cohesion can be established by such "preloaded" cohesin complexes [29], implying that DNA polymerases are able to move through cohesin rings. This hypothesis could explain how DNA replication would

[2]Co-first author
[3]Present address: Eucodis Bioscience GmbH, Viehmarktgasse 2/2a, 1030 Vienna, Austria
*Correspondence: peters@imp.ac.at
This is an open access article under the CC BY license (http://creativecommons.org/licenses/by/3.0/).

automatically lead to entrapment of sister chromatids inside cohesin's ring structure, but direct evidence for this "replication-through-the-ring" model is missing.

To understand how cohesin is loaded onto DNA and establishes cohesion, we have generated human cohesin complexes in wild-type (WT), nonacetylatable, and ATP hydrolysis-deficient forms and have analyzed their properties as purified complexes, in *Xenopus* egg extracts, and by expression in HeLa cells. The results from these experiments provide further support for the hypothesis [10, 11] that cohesin loading occurs in two steps in which cohesin is first recruited to DNA by the loading complex and subsequently entraps DNA in an ATPase-dependent manner. Unlike previously proposed [25], cohesin's ATPase activity is not detectably altered by Smc3 acetylation. Unexpectedly, however, the opposite is the case. Smc3 acetylation is strictly dependent on cohesin's ability to hydrolyze ATP, both in vivo and in vitro. Because ATP hydrolysis is essential for entrapment of DNA inside the cohesin ring, our results indicate that cohesin acetylation is coupled to the loading of cohesin onto DNA. We discuss the implications of this finding, namely that cohesion establishment during DNA replication may not only depend on Smc3 acetylation but also on de novo loading of cohesin onto DNA.

Results

Recombinant Human Cohesin Complexes Are Functional ATPases

To guide the generation of human cohesin mutants, we performed in silico modeling of the human Smc1 and Smc3 ATPase domain using a yeast Smc1 crystal structure as a template [3]. As expected, the resulting model indicated that the signature motif of Smc1 contacts the Walker A and Walker B motifs of Smc3 and vice versa to form two composite ATP binding sites. At these sites, the Walker A and Walker B motifs are predicted to be required for ATP binding and hydrolysis, respectively [7]. The model also confirmed [18, 19] that these sites are in proximity to Smc3's acetylation sites K105 and K106 (Figure 1A).

To characterize the ATPase activity of human cohesin, we expressed dimeric (Smc1-Smc3) and trimeric (Smc1-Smc3-Scc1) complexes in Baculovirus-infected insect cells, purified these complexes by tandem affinity purification, and analyzed their composition by SDS-PAGE and silver staining (Figure 1B). ATPase thin-layer chromatography assays revealed that over time, trimeric complexes hydrolyzed more ATP than dimeric complexes (Figure 1C), consistent with the previous observation that the C-terminal winged helix of Scc1 stimulated the ATPase activity of yeast dimeric cohesin [30]. In both complexes, ATP hydrolysis was caused by Smc1 and Smc3 because mutation of K38 in the Walker A motif of Smc1 and Smc3 to alanine ("KA") reduced ATP hydrolysis (Figure 1D; Figure S1A available online).

At a concentration of 50 μM ATP, WT trimers hydrolyzed 2.4 ± 0.7 mol ATP per mol cohesin complex per minute. To measure kinetic constants, we performed substrate titration experiments with WT forms and KA mutants, obtained from three independent purifications (Figures 1E and 1F), and found that WT trimers exhibited a Michaelis-Menten constant (K_M) of 467 ± 55 μM. When incubating trimeric cohesin with an ATP concentration close to this value, we consequently observed that the ATP hydrolysis rate increased by one order of magnitude (Figure S1B).

The observed ATP hydrolysis rate of the Smc1-Smc3 dimer was indistinguishable from "background" levels at low enzyme concentrations but could be well distinguished at concentrations above 200 nM (Figure S1C). We therefore used a 5-fold higher concentration for the dimer than for the trimer in a substrate titration experiment. Under these conditions, WT dimers hydrolyzed 0.3 mol ATP per mol complex per minute (Figures 1G and S1B). The specific activities of the human dimers and trimers are similar to the ones reported for the corresponding yeast complexes (2.1 moles of ATP per mol complex per minute for budding yeast cohesin [30]; 3.7 min^{-1} for fission yeast cohesin [10]).

To test whether the ATPase activity of human cohesin is required for cohesion, we used a *Xenopus* egg extract system. We immunodepleted endogenous *Xenopus* cohesin from interphase extract with SA1 and SA2 antibodies, added purified human tetrameric cohesin (Smc1-Smc3-Scc1-SA1; Figures S1D and S1E), allowed the extract to replicate sperm chromatin, and then triggered chromosome condensation by addition of nondegradable cyclin B. Immunofluorescence microscopy revealed that normal sister chromatid cohesion was observed in 70% of chromosome spreads from control-depleted extracts, whereas cohesion defects were observed in 80% of chromosome spreads following depletion of endogenous cohesin (Figure 1H). Adding back WT, but not KA mutant, cohesin prevented these cohesion defects (Figures 1H and 1I). These results show that the recombinant cohesin complexes generated here are able to mediate cohesion and indicate that their ATPase activity is essential for this function.

Cohesin ATPase Mutants Associate Transiently with Chromatin

Because the function of cohesin's ATPase activity has so far only been analyzed in yeast, we analyzed the properties of cohesin ATPase mutants in human cells. We first tested if point mutations in the Walker A (K38A or KA), signature motif (S1116R or SR), or Walker B (E1144Q or EQ) motifs of Smc3 are sufficient to abolish cohesin's ATPase activity, as is the case for yeast cohesin [30]. For this purpose, we purified cohesin trimers containing Smc1, Scc1, and either WT or mutated forms of Smc3 from Baculovirus-infected insect cells (Figure 2A). None of the three resulting cohesin "hemimutants" showed detectable ATPase activity (Figure 2B), confirming that mutation of Smc3's ATP binding sites is sufficient to prevent ATP hydrolysis also at Smc1's NBD [30]. This finding enabled us to analyze the behavior of cohesin ATPase mutants by expressing the above-mentioned Smc3 mutants in HeLa cells and following their behavior in fluorescence recovery after photobleaching (FRAP) experiments, without having to coexpress mutated forms of Smc1 and having to deplete endogenous Smc1 and Smc3. For these experiments, we modified a bacterial artificial chromosome (BAC) containing the mouse *Smc3* locus and a C-terminal localization and affinity purification (LAP) tag by introducing the same point mutations as described above. The Smc3 mutants were stably expressed in HeLa cells at levels close to or below endogenous human Smc3, they assembled with endogenous subunits into cohesin complexes (Figure S2), and the GFP moiety of the LAP tag was used for FRAP analyses.

Previous FRAP experiments had indicated that cohesin diffuses throughout the nucleus and cytosol and binds to unreplicated DNA with a residence time in the range of minutes [31]. This "dynamic binding mode" is thought to be the result of

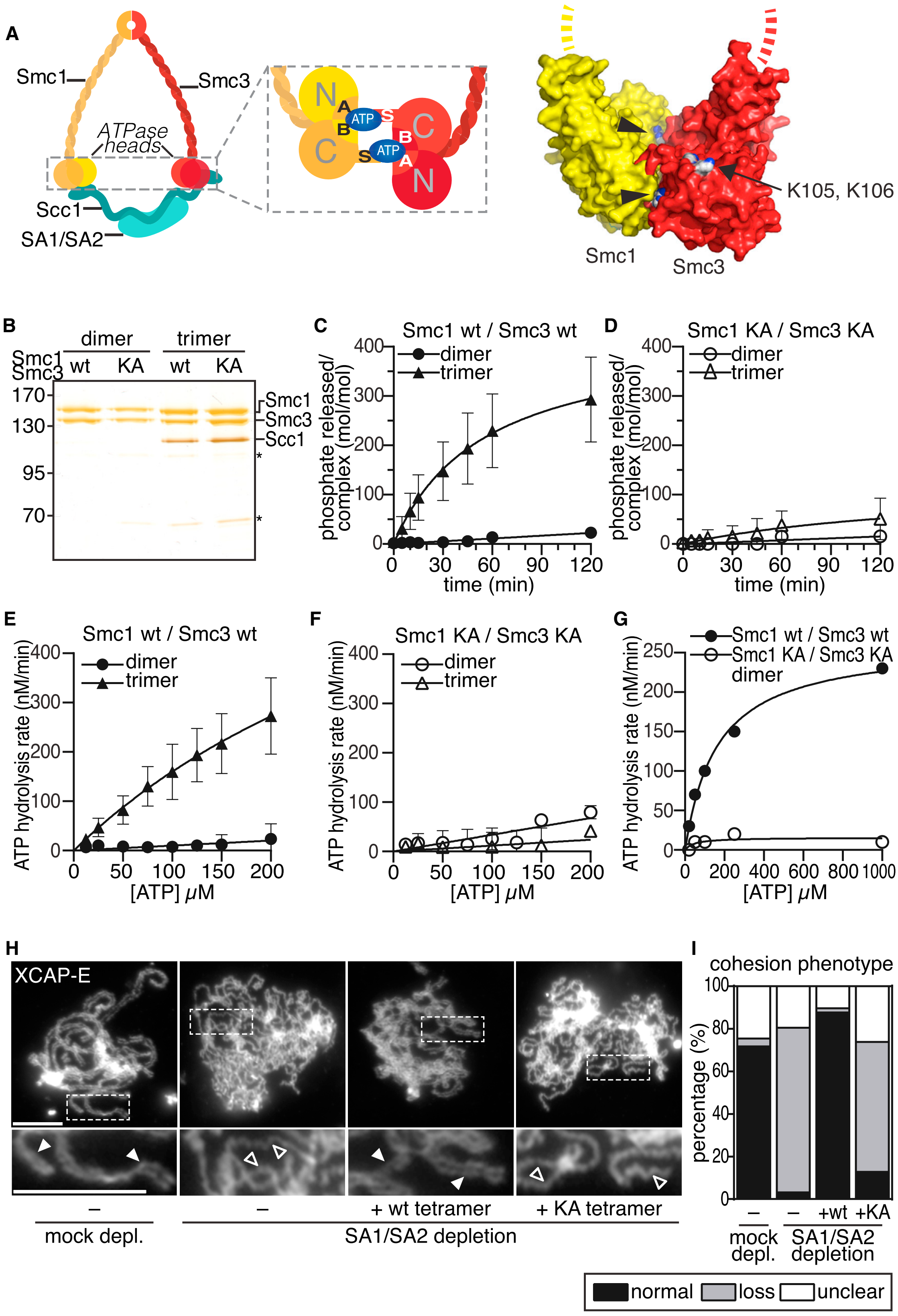

Figure 1. Reconstitution and Functional Characterization of Human Cohesin

(A) Schematic model of a cohesin tetramer. Enlarged view indicates how Walker A (A), signature (S), and Walker B (B) motifs cooperate to bind and hydrolyze ATP. The model structure on the right shows the proximity between Smc3 acetylation (K105 and K106; arrow) and ATP binding sites (arrowheads) (based on Protein Data Bank 1W1W; [3]).

(legend continued on next page)

continuous loading of cohesin onto chromatin and subsequent release by Wapl-mediated opening of the exit gate [5, 12, 13, 15, 17]. To measure the chromatin-binding abilities of the ATPase mutants, we simplified the analysis of FRAP experiments by synchronizing cells in G1 phase and by photobleaching only within the nucleus (Figure 2C). To determine the diffusion coefficient of cohesin that is not bound to chromatin, we also analyzed the FRAP redistribution kinetics of cohesin in mitotic cells, where most cohesin is released from chromosomes by the Wapl-dependent "prophase pathway" [16, 17, 31, 32]. We determined a mean (±SEM) diffusion coefficient for WT Smc3-LAP of 2.96 ± 0.19 $\mu m^2/s$ (n = 10) after data normalization [33] and integrated this value in the FRAP analysis as a diffusion parameter.

Consistent with previous reports, WT Smc3-LAP in G1 phase did not turn over completely on chromatin within our experimental time frame, indicating that more than 40% of nuclear cohesin was bound to chromatin (Figure 2D). However, in contrast to previous findings [31], our data could not be explained by a single population of cohesin being associated with chromatin (dotted line in Figure 2D). Instead, a sum of two exponential functions (dashed black line in Figure 2D) representing two populations of cohesin with reduced fluorescence recovery was required to describe the data accurately. This indicates that cohesin can interact with unreplicated chromatin in two different modes: one that results in dissociation of cohesin from chromatin within less than 1 min, and another one that results in a residence time of several minutes. The second interaction mode corresponds to the "dynamic binding mode" previously described by Gerlich et al. [31], whereas we refer to the first one as "transient binding mode." We suspect that the transient binding mode was not detected by Gerlich et al. [31] because in this study, the measurement of fluorescence recovery was only started 1 min after photobleaching.

When we analyzed Smc3 mutants by FRAP, we observed that the Walker A, the signature motif, and the Walker B motif mutant showed faster redistribution kinetics than WT cohesin, indicating that their residence times on DNA were reduced (Figure 2E). However, the fluorescence signals of the ATPase mutants did not recover to the same degree as mitotic WT cohesin (Figure 2D), implying that the ATPase mutants were still able to interact with chromatin. Further analysis of the recovery kinetics indicated that the ATPase mutants interacted with chromatin predominantly through the transient interaction mode, whereas the dynamic binding mode was greatly reduced (Figures 2F and 2G).

These results indicate that cohesin shows two types of chromatin association in G1 phase: (1) a transient association in the range of tens of seconds that does not depend on cohesin's ATPase activity, and (2) a more long-lasting "dynamic" type of chromatin interaction that depends on cohesin's ability to hydrolyze ATP.

The Chromatin Association of Cohesin ATPase Mutants Is Regulated by the Loading Complex, but Not by Wapl

We next analyzed if the transient chromatin interaction of the ATPase mutants depends on the cohesin loading complex. For this purpose, we synchronized the different HeLa cell lines in G1 phase (Figure 3A) and transfected cells with small interfering RNA (siRNA) specific for Scc4/MAU2 or with control siRNA. Subsequently, we analyzed the levels of Smc3-LAP on chromatin by immunofluorescence microscopy in cells from which soluble proteins had been removed by pre-extraction (Figure 3B), by immunoblotting after separation of cell lysates into chromatin and supernatant fractions (Figure 3C). We also analyzed asynchronously proliferating cells by FRAP (Figures 3D and 3E). Although the FRAP experiments in Figure 2F had indicated that similar proportions of WT and ATPase mutant cohesin are associated with chromatin, we observed in both immunofluorescence microscopy and immunoblotting experiments smaller amounts of the ATPase mutants than WT cohesin on chromatin. We suspect that the reduced levels of the ATPase mutants on chromatin are caused by their short chromatin residence time, which may have led to their partial dissociation from chromatin during sample preparation. Importantly, the low levels of all three ATPase mutants on chromatin were further reduced in cells depleted of Scc4/MAU2 (Figures 3B and 3C), indicating that the association of these mutants with chromatin still depends on the cohesin loading complex. FRAP analysis confirmed this notion because Scc4 depletion increased the recovery time of both WT Smc3 and Smc3 KA by increasing the fraction of freely diffusing cohesin (Figures 3D and 3E). Similar observations have been made in yeast where the chromatin association of an Smc1 ATP hydrolysis mutant (E1158Q) depends on Scc2 [11].

It has been proposed that yeast cohesin is first recruited to DNA by the loading complex and that cohesin's ATPase activity is subsequently needed to entrap cohesin inside its ring structure [10, 11]. We tested predictions made by this hypothesis by analyzing the behavior of human cohesin ATPase mutants in cells depleted of Wapl. In such cells, WT cohesin accumulates on chromatin [15, 17], presumably because these cohesin complexes entrap DNA inside their ring structure but cannot be released from DNA again because Wapl would be needed to open their exit gate [5, 12, 13]. However, if cohesin ATPase mutants are deficient in the step that entraps DNA inside the cohesin ring, Wapl depletion should not lead to a stabilization of these mutants on chromatin. To test this prediction, we depleted Wapl by RNAi (Figure S3A) in HeLa cells expressing WT or KA mutant Smc3-LAP and analyzed the behavior of these proteins by FRAP (Figures 3D and 3E).

Whereas the recovery of fluorescent WT cohesin was greatly reduced following Wapl depletion, the recovery of ATPase mutant cohesin was not and was instead slightly increased (Figure 3D). Further analysis of the FRAP kinetics revealed that Wapl depletion increased the amount of WT cohesin on

(B) Purified WT and Walker A mutant (KA) dimeric and trimeric (with Scc1) cohesin complexes were analyzed by silver staining.
(C and D) Time course quantification of phosphate released after incubation of purified complexes with ATP [γ-^{32}P] to determine specific enzymatic activities. Error bars denote SD (n = 3).
(E and F) Substrate dose-response quantification of purified complexes to measure ATP hydrolysis rates. Error bars denote SD (n = 3).
(G) Substrate dose response of cohesin dimers at high enzyme concentration to quantify ATP hydrolysis rates.
(H) XCAP-E staining of *Xenopus* extracts after depletion (depl.) of SA1/SA2, addition of purified human cohesin tetramers, and assembly of mitotic chromosomes to determine the degree of sister chromatid cohesion. Closed arrowheads indicate paired, open arrowheads unpaired sister chromatids. Scale bars represent 10 μm. The efficiency of cohesin depletion in this experiment is shown in Figure 5A.
(I) Chromosome cohesion phenotype after XCAP-E staining as depicted in (H) was quantified (n $\geq$ 155 per condition).
See also Figure S1.

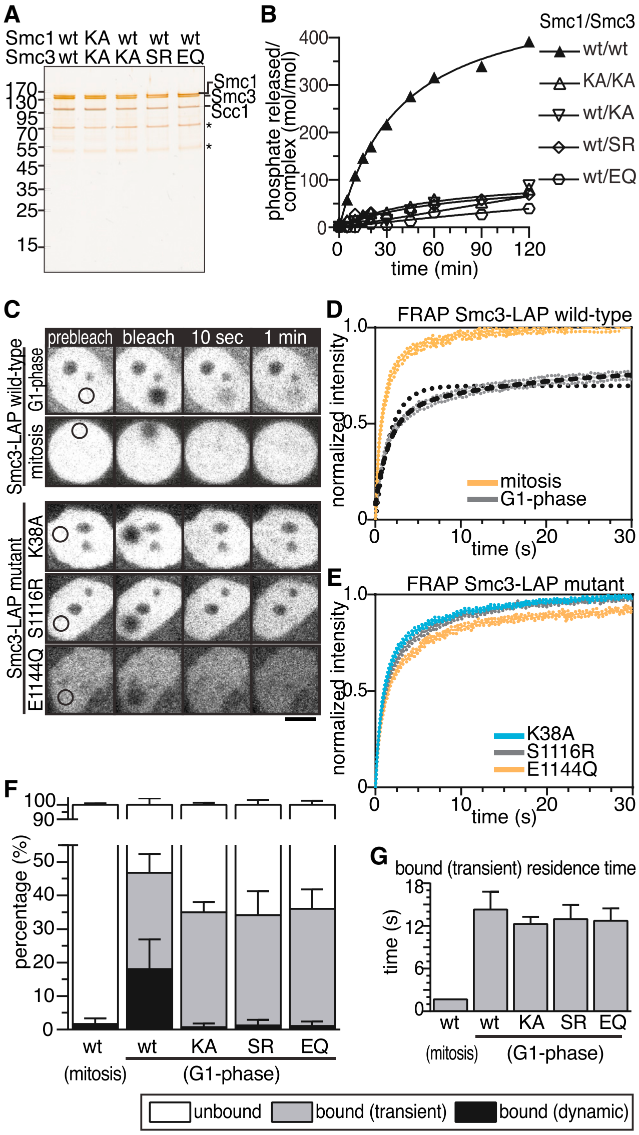

Figure 2. Cohesin ATPase Mutants Associate Transiently with Chromatin

(A) Silver staining of purified trimeric cohesin complexes after mutation of ATPase sites in Smc3 or Smc1/Smc3 subunits (KA, Walker A mutant; SR, signature mutant; EQ, Walker B mutant). Asterisks denote unidentified proteins.

(B) Time course quantification of phosphate released from ATPase reactions with cohesin mutated at one or both ATPase-active sites.

(C) Still images of FRAP experiments with HeLa cells expressing mouse Smc3-LAP. Cells were synchronized in G1 phase or were in mitosis. Black circles illustrate site of bleaching (2 μm radius). Scale bar represents 10 μm.

(D) Quantification of fluorescent signals after normalization from Smc3-LAP WT cells treated as in (C). Dotted line illustrates fitted curve using single-phase association; dashed line shows fitted curve with a two-phase association function.

(E) Quantification of ATPase mutant FRAP signals.

(F) Quantification of the cohesin distribution within the nucleus as freely diffusing (unbound), transiently chromatin-bound, and dynamically chromatin-bound populations.

(G) Calculated residence time of transiently chromatin-bound cohesin pools.

Error bars in (D)–(G) denote SEM ($n \geq 20$ per condition in G1 phase, $n = 10$ in mitosis). See also Figure S2.

chromatin, whereas the amounts of the ATPase mutant cohesin on chromatin were slightly reduced under these conditions (Figures 3E and S3B). These findings are consistent with the hypothesis that cohesin is first recruited to DNA by the cohesin loading complex and subsequently entraps DNA in a step that depends on cohesin's ATPase activity. We do not know why Wapl depletion slightly increases the recovery of the Smc3-LAP KA mutant in FRAP assays, but it is possible that this is an indirect effect of chromatin compaction that is known to be caused by stabilization of WT cohesin on chromatin in Wapl-depleted cells ([15]; Figure S3B).

Cohesin Acetylation Does Not Influence Its ATPase Activity

Because Smc3 acetylation occurs in the proximity of cohesin's ATP binding sites (Figure 1A; [18, 19]) and has been proposed to diminish cohesin's ATPase activity [25], we attempted to measure the ATPase activity of acetylated cohesin. To generate acetylated cohesin, we first incubated recombinant cohesin complexes with purified human Esco1 and its cofactor, acetyl-coenzyme A (CoA). Although the Esco1 enzyme used in these experiments was able to acetylate cohesin associated with *Xenopus* sperm chromatin [23], we were unable to detect Smc3 acetylation in the purified system (see Figure 5B). We therefore isolated cohesin dimers and trimers from insect cells in which Esco1 had been coexpressed. Under these conditions, Smc3 acetylation could be detected with an acetyl-specific Smc3 antibody [23] in the purified cohesin complexes (Figures S4A, S4B, S4D, and S4E). We estimate that in these samples, approximately half of the cohesin molecules had been acetylated (see legend for Figure S4B). However, the specific ATPase activity of these complexes did not differ significantly from the activity of cohesin samples in which no acetylation could be detected (Figures S4C and S4F).

We also generated cohesin complexes containing forms of Smc3 in which K105 and K106 had been mutated to glutamine (QQ), arginine (RR), or alanine (AA; Figures 4A and 4C). These three Smc3 mutants resemble acetylated Smc3 in its ability to bind sororin, which normally only interacts with acetylated but not with nonacetylated cohesin, implying that these mutants functionally mimic the acetylated form of Smc3 [23]. We therefore tested if cohesin dimers and trimers containing the QQ, RR, or AA mutants of Smc3 were altered in their specific ATPase activity. However, this was not the case (Figures 4B and 4D). Taken together, these results indicate that Smc3 acetylation has no major effect on cohesin's ATPase activity, at least under our assay conditions.

Cohesin's ATPase Activity Is Essential for Smc3 Acetylation

In the immunoprecipitation experiments shown in Figure S2, we had failed to detect acetylated Smc3 in the cohesin ATPase mutants expressed in HeLa cells, even though we had analyzed similar amounts of ATPase mutant and WT complexes by immunoblotting and had found that WT cohesin was clearly acetylated under these conditions. Furthermore, the ATPase mutant complexes contained little if any sororin, presumably due to the absence of acetylated Smc3 (Figure S2A). Similar results were obtained when WT or Walker A mutant (KA) tetrameric cohesin complexes were added to cohesin-depleted *Xenopus* egg extract and incubated with sperm DNA to initiate cohesin loading and DNA replication. After 120 min, chromatin-bound proteins were analyzed by immunoblotting. Although both WT and KA complexes bound to sperm chromatin, WT cohesin contained much more

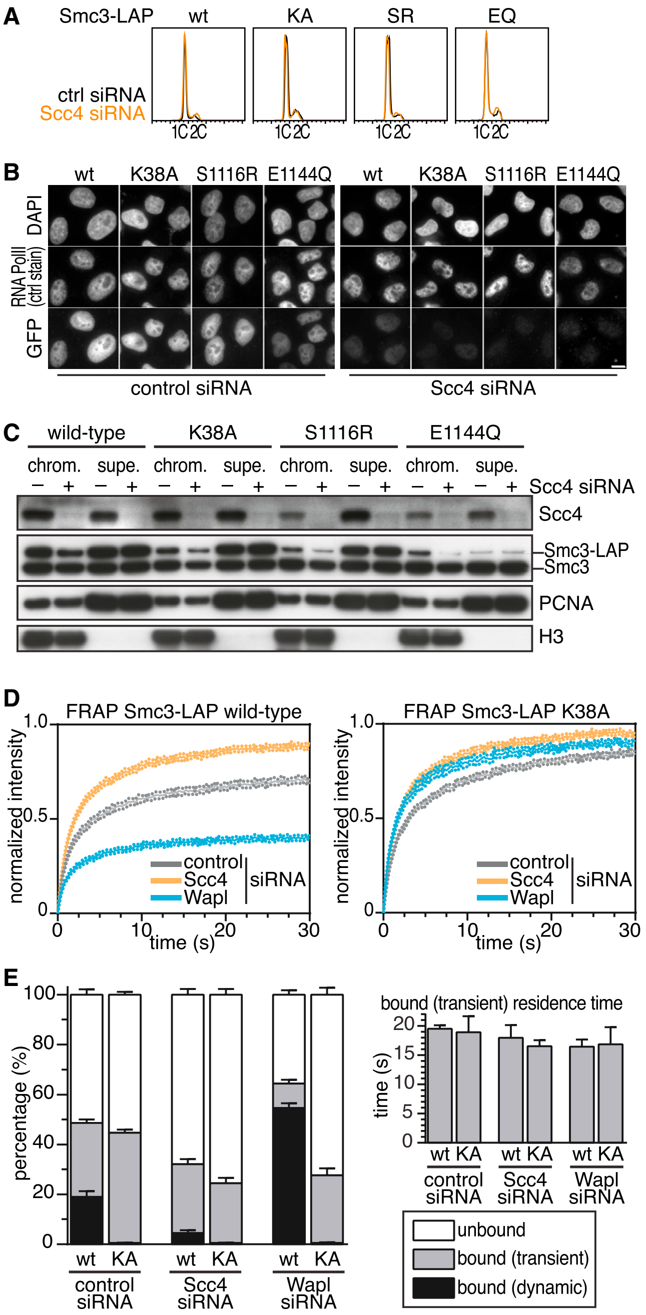

Figure 3. Chromatin Association of Cohesin ATPase Mutants Depends on Scc4, but Not on Wapl

(A) Fluorescence-activated cell-sorting profiles of cells synchronized in G1 phase after 48 hr Scc4 depletion and stained with propidium iodide. ctrl, control.

(B) Immunofluorescence microscopy experiment showing localization of Smc3-LAP on chromatin after RNAi and pre-extraction before fixation. DAPI was used to stain DNA, RNA polymerase II (PolII) staining was used as a signal intensity control, and GFP antibody was used to detect Smc3-LAP. Scale bar represents 10 μm.

(C) Chromatin (chrom.) and soluble (supe.) fractions of cells from (A) and (B) were analyzed by immunoblotting.

(D) Quantification of fluorescent signals in FRAP experiments after control, Scc4, or Wapl depletion for 72 hr.

(E) Quantification of experiments in (D) to measure unbound, transiently bound, and dynamically bound pools of cohesin and the calculated residence time of the transiently bound state.

Error bars in (D) and (E) denote SEM (n ≥ 13 per condition). See also Figure S3.

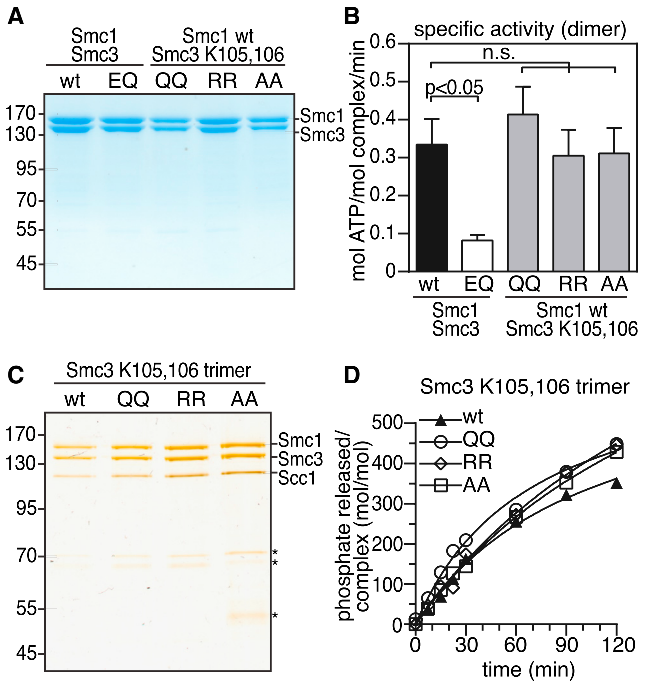

Figure 4. Smc3 Acetylation Does Not Detectably Affect Cohesin's ATPase Activity

(A) Coomassie staining of cohesin dimers mutated at Walker B (Smc1 E1157Q and Smc3 E1144Q) or acetylation sites (Smc3 K105 and K106).

(B) Comparison of specific activities of WT and mutant dimeric cohesin complexes in (A). Error bars denote SEM (n ≥ 8).

(C) Silver staining of purified cohesin trimers mutated at lysines 105 and 106. Asterisks denote unidentified proteins.

(D) Comparison of hydrolysis rates for cohesin complexes from (C).

See also Figure S4.

acetylated Smc3 than the ATPase-deficient complex (Figure 5A, compare lanes 9 and 11).

These results indicate that cohesin ATPase mutants cannot be acetylated. This deficiency could be an indirect consequence of the short residence time of the ATPase mutants on chromatin. Alternatively, cohesin's ATPase activity could be directly required for the acetylation reaction. To test the latter possibility, we analyzed if purified Esco1 (Figure S5A) could acetylate recombinant cohesin complexes in vitro under conditions where these complexes can hydrolyze ATP. As mentioned above, no Smc3 acetylation could be detected when cohesin dimers were incubated with Esco1 and acetyl-CoA. However, when the same reactions were carried out in the presence of ATP, Smc3 acetylation was readily detectable (Figure 5B). ATP did not have this effect when cohesin Walker A mutants were used as a substrate (Figure 5B), indicating that ATP enabled Smc3 acetylation by affecting cohesin and not Esco1.

When we used cohesin dimers as substrates, ADP promoted Smc3 acetylation similarly well as ATP (Figure 5B), but when we used cohesin trimers, much more Smc3 acetylation was observed in the presence of ATP than of ADP (Figure 5C). Together with the finding that trimers have a higher specific ATPase activity than dimers (Figure 1C), this observation implies that ATP hydrolysis supports Smc3 acetylation more efficiently than ADP binding. Consistent with this interpretation, we found in a time course experiment that ATP enabled the acetylation of cohesin trimers much more rapidly than ADP (Figure 5D). Furthermore, we observed that the

nonhydrolyzable ATP analog adenylylimidodiphosphate (AMP-PNP) did not promote Smc3 acetylation above "background" levels (Figure 5C), although it efficiently inhibited ATP hydrolysis by WT cohesin (Figure S5B), suggesting that AMP-PNP bound to these cohesin complexes. Similarly, we observed that trimeric complexes mutated at Smc3's Walker B motif (EQ) were not acetylated in the presence of ATP (Figure 5E), although these complexes are predicted to bind ATP (but to be unable to hydrolyze it [7]).

To further test if cohesin's ATPase activity is required for Smc3 acetylation, we also tested if mutation of Smc1's NBD abrogates Smc3 acetylation (note that all of the above experiments had been carried out with Smc3 hemimutants or Smc1-Smc3 double mutants). As expected, mutation of the Walker A (K38A) and signature (S1129R) motifs in Smc1 reduced Smc3 acetylation of cohesin trimers, but mutation of Smc1's Walker B motif (E1157Q) had no detectable effect on ATP-dependent Smc3 acetylation (Figures S5C–S5F). However, ATPase assays revealed that the Smc1 EQ hemimutant was still able to hydrolyze ATP at a reduced rate (Figure S5D). This Walker B mutation is thought to prevent ATP hydrolysis but not ATP binding at Smc1's NBD, raising the possibility that ATP binding at Smc1's NBD is sufficient to trigger ATP hydrolysis by the Smc3 subunit. Consistent with this interpretation, a point mutation in the Walker B motif of Smc3 (EQ) abrogated the activity of the Smc1 Walker B mutant (Figure S5E). Importantly, this double mutant could not be acetylated (Figure S5B). The ability of Esco1 to acetylate Smc3 therefore correlates with the ability of cohesin to hydrolyze ATP.

Our experiments in which we had coexpressed cohesin and Esco1 in insect cells had indicated that Smc3 acetylation does not significantly alter cohesin's ATPase activity (see Figure S4). To further test this notion, we also incubated purified cohesin trimers with Esco1 and ATP in either the absence or presence of acetyl-CoA, reisolated cohesin by immunoprecipitation, and measured its ATPase activity (Figure 5F). Also in this experiment, the ATPase activities of both samples were undistinguishable (Figure 5H), despite the fact that Smc3 acetylation occurred in the presence of Esco1, ATP, and acetyl-CoA, but not in the absence of acetyl-CoA (Figure 5G). Also under these conditions, the ATPase activity could be attributed to cohesin

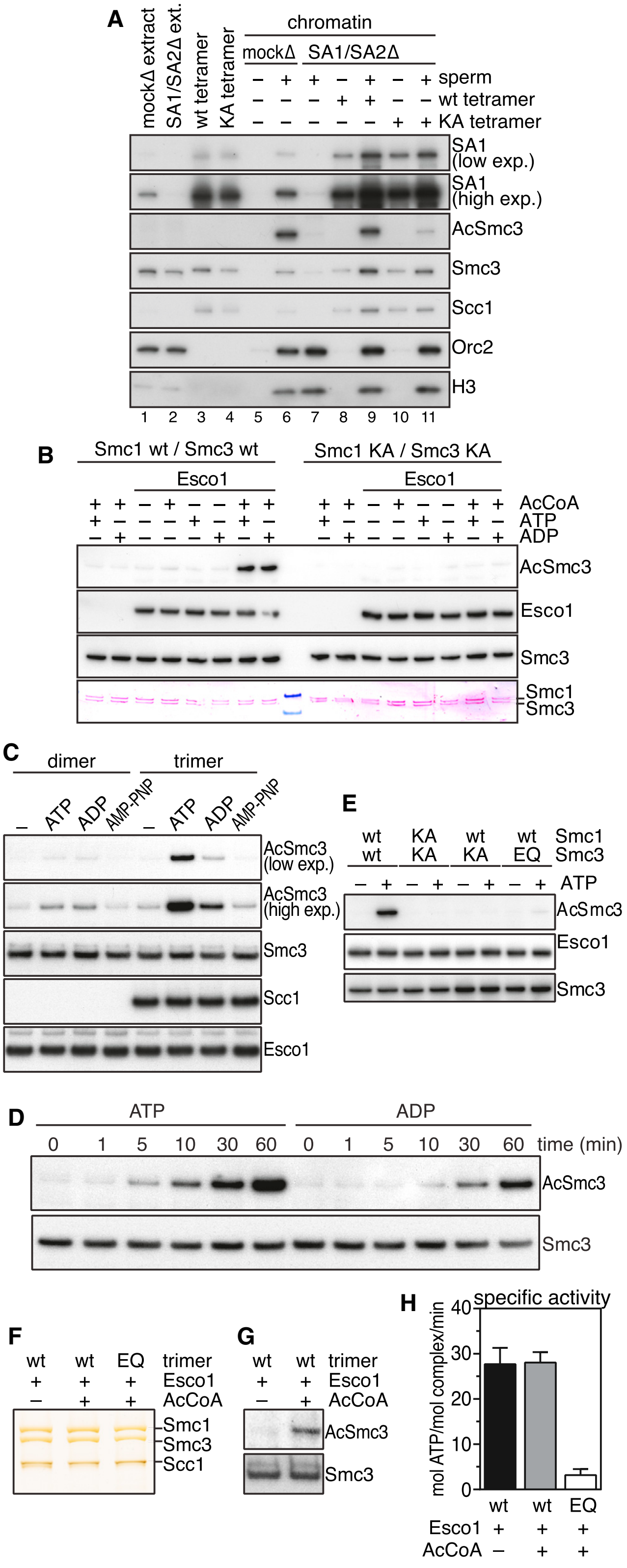

Figure 5. Cohesin's ATPase Activity Is Required for Smc3 Acetylation

(A) *Xenopus* extracts (ext.) were immunodepleted with an antibody against SA1/SA2 (lane 2) and supplemented with human cohesin complexes (lanes 3 and 4) before sperm addition (see also Figures 1H and S1D). Two hours later, chromatin fractions (lanes 6, 7, 9, and 11) and samples without sperm addition (to control for the dependence of cohesin pelleting on chromatin association) were analyzed by immunoblotting. Please note that SA1/SA2 depletion does not lead to complete codepletion of Smc3 (lane 2) because these extracts also contain Smc1-Smc3 heterodimers that are not bound to SA1 or SA2 [34, 35]. However, only a little of the Smc1-Smc3 heterodimer that remains in the extract after SA1/SA2 immunodepletion associates with chromatin (lane 7), and the heterodimer is not expected to contribute to sister chromatid cohesion (see Figure 1H). exp., exposure.

(B) Purified dimeric cohesin complexes were incubated with human Esco1, acetyl-CoA, and ATP or ADP (see also Figure S5A). Acetylation and protein levels were analyzed by western blotting and Ponceau staining.

(C) Cohesin dimers and trimers were subjected to the acetylation reaction in the presence of ATP, ADP, or AMP-PNP.

(D) Trimeric cohesin complexes were subjected to the acetylation reaction in the presence of ATP or ADP, and the degree of acetylation was assayed at the indicated time points.

(E) Trimeric cohesin complexes mutated at one or both ATPase domains were assayed in the acetylation reaction in the presence or absence of ATP.

(F) Silver staining of purified cohesin trimers (WT or EQ mutant) after incubation with Esco1 and ATP in the presence or absence of acetyl-CoA for 60 min at 37°C.

(G) WT cohesin trimers from (F) were analyzed by immunoblotting for the presence of acetylated Smc3.

(H) ATP hydrolysis quantification of cohesin trimers in (F) after incubation with 400 μM ATP. Error bars denote SD (n = 3).

See also Figure S5.

because a cohesin trimer in which both Smc1 and Smc3 had been mutated in their Walker B motifs (EQ) showed much less ATP hydrolysis activity (Figure 5H).

Discussion

Cohesin, first discovered as a protein complex essential for sister chromatid cohesion, is now known to carry out a variety of important functions in both proliferating and postmitotic cells, ranging from DNA repair to chromatin organization and gene regulation. Cohesin mediates all of these functions by interacting with DNA, presumably by topologically entrapping DNA inside its ring structure [4]. Understanding how cohesin entraps DNA and how this interaction is regulated is therefore of great importance.

Previous FRAP experiments had revealed that cohesin can interact with chromatin in two different ways: a dynamic and a stable binding mode [31]. The dynamic binding mode is thought to be the result of continuous cohesin loading and release reactions, mediated by the loading complex and Wapl, respectively, and has been proposed to contribute to chromatin organization and gene regulation [12, 15]. The stable binding mode occurs only during and after DNA replication, depends on Smc3 acetylation and sororin binding, and is thought to be required for mediating cohesion from S phase until mitosis [23, 36]. The FRAP data presented here provide direct evidence for a third, much more transient binding mode with which cohesin can interact with chromatin in the range of seconds. This binding mode might correspond to transient cohesin-chromatin interactions that have previously been observed in *D. melanogaster* [37]. Several observations indicate that this transient interaction represents an intermediate step in the loading reaction. Cohesin ATPase mutants retain the ability to interact with chromatin transiently in a manner that depends on the loading complex, but unlike WT cohesin, these mutants fail to associate with chromatin for longer periods of time in the absence of Wapl. These results provide further support for the hypothesis proposed by Hu et al. [11] and Murayama and Uhlmann [10] that cohesin is initially recruited to DNA via the cohesin loading complex, resulting in a transient cohesin-chromatin interaction, and that subsequent ATP hydrolysis by cohesin is needed to entrap DNA inside the cohesin ring, resulting in the dynamic binding mode. How ATP hydrolysis at cohesin's ATP binding sites could lead to separation of the hinge domains of Smc1 and Smc3 at the other "end" of cohesin remains a mystery. It has been speculated that ATP hydrolysis might induce a conformational switch that is transmitted via the coiled coils of Smc1 and Smc3 to the hinge regions [6], possibly assisted by multiple contacts of the loading complex with the cohesin ring [10].

The proximity of cohesin's ATP binding sites to the acetylated lysine residues on Smc3 has led to speculations about possible roles of Smc3 acetylation in controlling cohesin's ATPase activity [19]. Consistent with such a role, it has been observed that dominant-negative effects of a yeast Smc3 ATPase mutant could be similarly reduced by mutating its acetyl or ATP binding sites [25]. However, in our biochemical assays, we were unable to detect effects of Smc3 acetylation or of mutations introduced into the acetylation sites on cohesin's ATPase activity. Although we cannot exclude the existence of such effects in the cellular context, we suspect that Smc3 acetylation stabilizes cohesin on chromatin by other mechanisms than inhibiting cohesin's ATPase activity, namely by recruitment of sororin and inactivation of Wapl.

Unexpectedly, we observed that the opposite was the case: Smc3 acetylation was strictly dependent on cohesin's ATPase activity in both HeLa cells and *Xenopus* egg extracts. Similar observations have been made for cohesin ATPase mutants in yeast, but in this case, the absence of acetylation has been attributed to the transient association of these mutants with chromatin [38] where Smc3 acetylation occurs [23]. However, our finding that Smc3 acetylation also depends on cohesin's ATPase activity in a reconstituted system containing recombinant cohesin and Esco1 implies that the short residence time of these mutants on chromatin is not the only and possibly not the main reason why Smc3 does not become acetylated in these mutants. Instead, our results indicate that cohesin's ability to hydrolyze ATP into ADP is a prerequisite for Smc3 acetylation. Although we found that ADP can also support Smc3 acetylation to some degree, we suspect that under physiological conditions, it is the process of ATP hydrolysis that enables Smc3 acetylation because in cells, the concentration of ATP is much higher than the concentration of ADP, and because ATP supported the acetylation of trimeric cohesin much better and more rapidly than ADP. Because we have so far not been able to generate recombinant active Esco2, we do not know if Smc3 acetylation by this enzyme also depends on cohesin's ATPase activity. However, we suspect that this is the case because Esco1 and Esco2 modify the same lysine residues on Smc3, and because the acetylation of ATPase mutants was strongly diminished in *Xenopus* egg extracts, which contain little if any Esco1 [22] and in which WT cohesin is therefore exclusively acetylated by Esco2.

Why is Smc3 acetylation dependent on cohesin's ATPase activity? It is plausible to think that ATPase activity converts cohesin into a conformation that makes it susceptible for acetylation. If this conformation can be maintained for longer periods of time, it is conceivable that cohesin loaded onto chromatin in G1 phase would become acetylated later during DNA replication to establish sister chromatid cohesion. Consistent with such a sequential model, it has been proposed that cohesin loaded onto chromatin before DNA replication is sufficient to mediate sister chromatid cohesion later in the cell cycle [29]. Alternatively, it is conceivable that normally, cohesion is established by cohesin complexes that are loaded de novo onto chromatin during DNA replication. Such a scenario could explain in functional terms why Smc3 acetylation depends on cohesin's ATPase activity. According to this hypothesis, ATP hydrolysis would mediate entrapment of newly synthesized sister DNA molecules and would at the same time convert Smc3 into a state that is susceptible to acetylation (Figure 6). This modification would then, through a poorly understood process, lead to recruitment of sororin and inhibition of Wapl. These events would "lock" the exit gate and thus lead to the stable binding mode of cohesin that is required for sister chromatid cohesion. Although speculative, this hypothesis could explain why in *Xenopus* egg extracts both the loading complex and Esco2 are recruited to prereplicative complexes where DNA replication is initiated [39–42].

Supplemental Information

Supplemental Information includes five figures and Supplemental Experimental Procedures and can be found with this article online at http://dx.doi.org/10.1016/j.cub.2014.08.011.

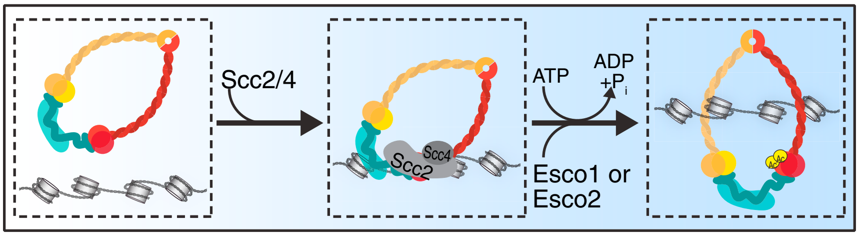

Figure 6. Cohesin's ATPase Activity Couples Cohesin Loading onto DNA with Smc3 Acetylation

Schematic model of cohesin loading and Smc3 acetylation. The cohesin loading complex composed of Scc2/NIPBL and Scc4/MAU-2 mediates association of cohesin with DNA. ATP binding and hydrolysis trigger chromatin entrapment and allow for Smc3 acetylation by Esco1 and presumably Esco2, thereby initiating cohesion establishment.

Author Contributions

V.B., R.L., P.J.H.i.t.V., I.F.D., E.K., G.P., and J.-M.P. designed experiments and interpreted data. V.B. and P.J.H.i.t.V. generated recombinant human cohesin. V.B. performed ATPase and acetylation assays. I.F.D. performed *Xenopus* experiments. E.K. generated Smc3-LAP-expressing cell lines and performed IP-MS experiments. R.L. performed FRAP and RNAi experiments. R.L., V.B., and J.-M.P. wrote the manuscript.

Acknowledgments

We thank D. Gerlich, all members of the J.-M.P. laboratory, and the IMP/IMBA Biooptics and MassSpec facilities for discussions and assistance. V.B. was supported by the European Molecular Biology Organization. Research in the J.-M.P. laboratory is supported by Boehringer Ingelheim, the Austrian Science Fund (FWF special research program SFB F34 "Chromosome Dynamics" and Wittgenstein award Z196-B20), the Austrian Research Promotion Agency (FFG, Laura Bassi Center for Optimized Structural Studies), the Vienna Science and Technology Fund (WWTF LS09-13), and the European Community's Seventh Framework Programme (FP7/2007-2013) under grant agreement 241548 (MitoSys).

Received: March 19, 2014
Revised: July 10, 2014
Accepted: August 6, 2014
Published: September 11, 2014

References

1. Tanaka, T., Fuchs, J., Loidl, J., and Nasmyth, K. (2000). Cohesin ensures bipolar attachment of microtubules to sister centromeres and resists their precocious separation. Nat. Cell Biol. *2*, 492–499.
2. Nasmyth, K., and Haering, C.H. (2009). Cohesin: its roles and mechanisms. Annu. Rev. Genet. *43*, 525–558.
3. Haering, C.H., Schoffnegger, D., Nishino, T., Helmhart, W., Nasmyth, K., and Löwe, J. (2004). Structure and stability of cohesin's Smc1-kleisin interaction. Mol. Cell *15*, 951–964.
4. Haering, C.H., Farcas, A.M., Arumugam, P., Metson, J., and Nasmyth, K. (2008). The cohesin ring concatenates sister DNA molecules. Nature *454*, 297–301.
5. Buheitel, J., and Stemmann, O. (2013). Prophase pathway-dependent removal of cohesin from human chromosomes requires opening of the Smc3-Scc1 gate. EMBO J. *32*, 666–676.
6. Gruber, S., Arumugam, P., Katou, Y., Kuglitsch, D., Helmhart, W., Shirahige, K., and Nasmyth, K. (2006). Evidence that loading of cohesin onto chromosomes involves opening of its SMC hinge. Cell *127*, 523–537.
7. Arumugam, P., Gruber, S., Tanaka, K., Haering, C.H., Mechtler, K., and Nasmyth, K. (2003). ATP hydrolysis is required for cohesin's association with chromosomes. Curr. Biol. *13*, 1941–1953.
8. Weitzer, S., Lehane, C., and Uhlmann, F. (2003). A model for ATP hydrolysis-dependent binding of cohesin to DNA. Curr. Biol. *13*, 1930–1940.
9. Ciosk, R., Shirayama, M., Shevchenko, A., Tanaka, T., Toth, A., Shevchenko, A., and Nasmyth, K. (2000). Cohesin's binding to chromosomes depends on a separate complex consisting of Scc2 and Scc4 proteins. Mol. Cell *5*, 243–254.
10. Murayama, Y., and Uhlmann, F. (2014). Biochemical reconstitution of topological DNA binding by the cohesin ring. Nature *505*, 367–371.
11. Hu, B., Itoh, T., Mishra, A., Katoh, Y., Chan, K.L., Upcher, W., Godlee, C., Roig, M.B., Shirahige, K., and Nasmyth, K. (2011). ATP hydrolysis is required for relocating cohesin from sites occupied by its Scc2/4 loading complex. Curr. Biol. *21*, 12–24.
12. Chan, K.L., Roig, M.B., Hu, B., Beckouët, F., Metson, J., and Nasmyth, K. (2012). Cohesin's DNA exit gate is distinct from its entrance gate and is regulated by acetylation. Cell *150*, 961–974.
13. Eichinger, C.S., Kurze, A., Oliveira, R.A., and Nasmyth, K. (2013). Disengaging the Smc3/kleisin interface releases cohesin from Drosophila chromosomes during interphase and mitosis. EMBO J. *32*, 656–665.
14. Lopez-Serra, L., Lengronne, A., Borges, V., Kelly, G., and Uhlmann, F. (2013). Budding yeast Wapl controls sister chromatid cohesion maintenance and chromosome condensation. Curr. Biol. *23*, 64–69.
15. Tedeschi, A., Wutz, G., Huet, S., Jaritz, M., Wuensche, A., Schirghuber, E., Davidson, I.F., Tang, W., cisneros, D.A., Bhaskara, V., et al. (2013). Wapl is an essential regulator of chromatin structure and chromosome segregation. Nature *501*, 564–568.
16. Gandhi, R., Gillespie, P.J., and Hirano, T. (2006). Human Wapl is a cohesin-binding protein that promotes sister-chromatid resolution in mitotic prophase. Curr. Biol. *16*, 2406–2417.
17. Kueng, S., Hegemann, B., Peters, B.H., Lipp, J.J., Schleiffer, A., Mechtler, K., and Peters, J.M. (2006). Wapl controls the dynamic association of cohesin with chromatin. Cell *127*, 955–967.
18. Rolef Ben-Shahar, T., Heeger, S., Lehane, C., East, P., Flynn, H., Skehel, M., and Uhlmann, F. (2008). Eco1-dependent cohesin acetylation during establishment of sister chromatid cohesion. Science *321*, 563–566.
19. Unal, E., Heidinger-Pauli, J.M., Kim, W., Guacci, V., Onn, I., Gygi, S.P., and Koshland, D.E. (2008). A molecular determinant for the establishment of sister chromatid cohesion. Science *321*, 566–569.
20. Zhang, J., Shi, X., Li, Y., Kim, B.J., Jia, J., Huang, Z., Yang, T., Fu, X., Jung, S.Y., Wang, Y., et al. (2008). Acetylation of Smc3 by Eco1 is required for S phase sister chromatid cohesion in both human and yeast. Mol. Cell *31*, 143–151.
21. Hou, F., and Zou, H. (2005). Two human orthologues of Eco1/Ctf7 acetyltransferases are both required for proper sister-chromatid cohesion. Mol. Biol. Cell *16*, 3908–3918.
22. Lafont, A.L., Song, J., and Rankin, S. (2010). Sororin cooperates with the acetyltransferase Eco2 to ensure DNA replication-dependent sister chromatid cohesion. Proc. Natl. Acad. Sci. USA *107*, 20364–20369.
23. Nishiyama, T., Ladurner, R., Schmitz, J., Kreidl, E., Schleiffer, A., Bhaskara, V., Bando, M., Shirahige, K., Hyman, A.A., Mechtler, K., and Peters, J.M. (2010). Sororin mediates sister chromatid cohesion by antagonizing Wapl. Cell *143*, 737–749.
24. Rankin, S., Ayad, N.G., and Kirschner, M.W. (2005). Sororin, a substrate of the anaphase-promoting complex, is required for sister chromatid cohesion in vertebrates. Mol. Cell *18*, 185–200.
25. Heidinger-Pauli, J.M., Onn, I., and Koshland, D. (2010). Genetic evidence that the acetylation of the Smc3p subunit of cohesin modulates its ATP-bound state to promote cohesion establishment in Saccharomyces cerevisiae. Genetics *185*, 1249–1256.
26. Chatterjee, A., Zakian, S., Hu, X.W., and Singleton, M.R. (2013). Structural insights into the regulation of cohesion establishment by Wpl1. EMBO J. *32*, 677–687.
27. Uhlmann, F., and Nasmyth, K. (1998). Cohesion between sister chromatids must be established during DNA replication. Curr. Biol. *8*, 1095–1101.
28. Losada, A., Hirano, M., and Hirano, T. (1998). Identification of *Xenopus* SMC protein complexes required for sister chromatid cohesion. Genes Dev. *12*, 1986–1997.
29. Lengronne, A., McIntyre, J., Katou, Y., Kanoh, Y., Hopfner, K.P., Shirahige, K., and Uhlmann, F. (2006). Establishment of sister chromatid cohesion at the S. cerevisiae replication fork. Mol. Cell *23*, 787–799.
30. Arumugam, P., Nishino, T., Haering, C.H., Gruber, S., and Nasmyth, K. (2006). Cohesin's ATPase activity is stimulated by the C-terminal Winged-Helix domain of its kleisin subunit. Curr. Biol. *16*, 1998–2008.

31. Gerlich, D., Koch, B., Dupeux, F., Peters, J.M., and Ellenberg, J. (2006). Live-cell imaging reveals a stable cohesin-chromatin interaction after but not before DNA replication. Curr. Biol. *16*, 1571–1578.
32. Waizenegger, I.C., Hauf, S., Meinke, A., and Peters, J.M. (2000). Two distinct pathways remove mammalian cohesin from chromosome arms in prophase and from centromeres in anaphase. Cell *103*, 399–410.
33. Ellenberg, J., Siggia, E.D., Moreira, J.E., Smith, C.L., Presley, J.F., Worman, H.J., and Lippincott-Schwartz, J. (1997). Nuclear membrane dynamics and reassembly in living cells: targeting of an inner nuclear membrane protein in interphase and mitosis. J. Cell Biol. *138*, 1193–1206.
34. Losada, A., Yokochi, T., Kobayashi, R., and Hirano, T. (2000). Identification and characterization of SA/Scc3p subunits in the *Xenopus* and human cohesin complexes. J. Cell Biol. *150*, 405–416.
35. Sumara, I., Vorlaufer, E., Gieffers, C., Peters, B.H., and Peters, J.M. (2000). Characterization of vertebrate cohesin complexes and their regulation in prophase. J. Cell Biol. *151*, 749–762.
36. Schmitz, J., Watrin, E., Lénárt, P., Mechtler, K., and Peters, J.M. (2007). Sororin is required for stable binding of cohesin to chromatin and for sister chromatid cohesion in interphase. Curr. Biol. *17*, 630–636.
37. Gause, M., Misulovin, Z., Bilyeu, A., and Dorsett, D. (2010). Dosage-sensitive regulation of cohesin chromosome binding and dynamics by Nipped-B, Pds5, and Wapl. Mol. Cell. Biol. *30*, 4940–4951.
38. Mishra, A., Hu, B., Kurze, A., Beckouët, F., Farcas, A.M., Dixon, S.E., Katou, Y., Khalid, S., Shirahige, K., and Nasmyth, K. (2010). Both interaction surfaces within cohesin's hinge domain are essential for its stable chromosomal association. Curr. Biol. *20*, 279–289.
39. Gillespie, P.J., and Hirano, T. (2004). Scc2 couples replication licensing to sister chromatid cohesion in *Xenopus* egg extracts. Curr. Biol. *14*, 1598–1603.
40. Higashi, T.L., Ikeda, M., Tanaka, H., Nakagawa, T., Bando, M., Shirahige, K., Kubota, Y., Takisawa, H., Masukata, H., and Takahashi, T.S. (2012). The prereplication complex recruits XEco2 to chromatin to promote cohesin acetylation in *Xenopus* egg extracts. Curr. Biol. *22*, 977–988.
41. Takahashi, T.S., Basu, A., Bermudez, V., Hurwitz, J., and Walter, J.C. (2008). Cdc7-Drf1 kinase links chromosome cohesion to the initiation of DNA replication in *Xenopus* egg extracts. Genes Dev. *22*, 1894–1905.
42. Takahashi, T.S., Yiu, P., Chou, M.F., Gygi, S., and Walter, J.C. (2004). Recruitment of *Xenopus* Scc2 and cohesin to chromatin requires the pre-replication complex. Nat. Cell Biol. *6*, 991–996.

Current Biology, Volume *24*

Supplemental Information

Cohesin's ATPase Activity Couples Cohesin Loading onto DNA with Smc3 Acetylation

Rene Ladurner, Venugopal Bhaskara, Pim J. Huis in 't Veld, Iain F. Davidson, Emanuel Kreidl, Georg Petzold, and Jan-Michael Peters

Supplemental Figures

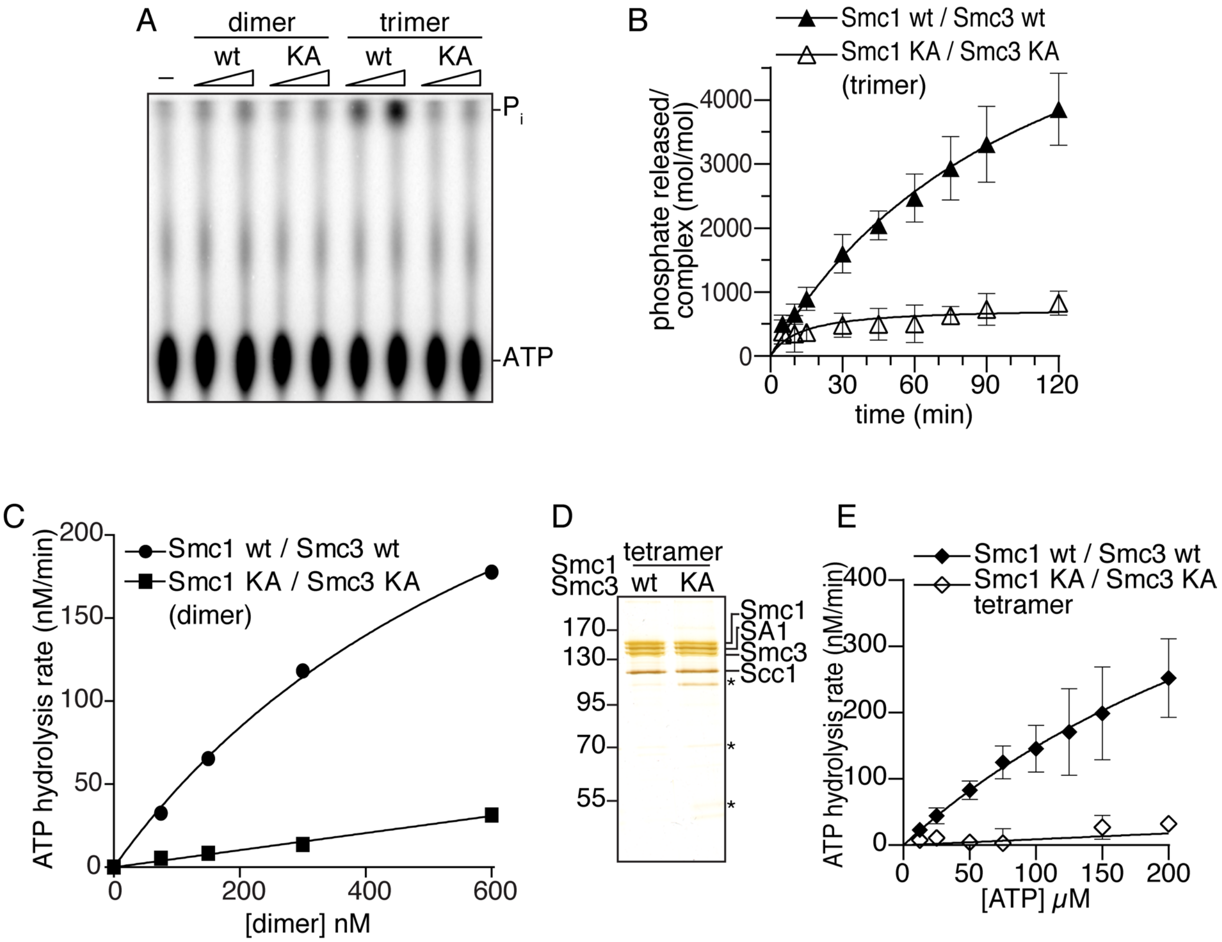

Figure S1, related to Figure 1. Reconstitution and functional characterization of human cohesin

(A) Thin-layer chromatography-autoradiography separated radiolabeled substrates and products of ATP hydrolysis reactions as indicated.
(B) Time course quantification of phosphate released after incubation of purified trimeric cohesin complexes with 400 μM ATP. Error bars denote standard deviation (n = 3).
(C) ATP hydrolysis rate quantification of dimeric cohesin as a function of enzyme concentration.
(D) Purified tetrameric cohesin complexes were analyzed by silver staining. Asterisks denote unidentified proteins.
(E) Substrate dose response quantification of purified complexes to measure ATP hydrolysis rates. Error bars denote standard deviation (n = 2).

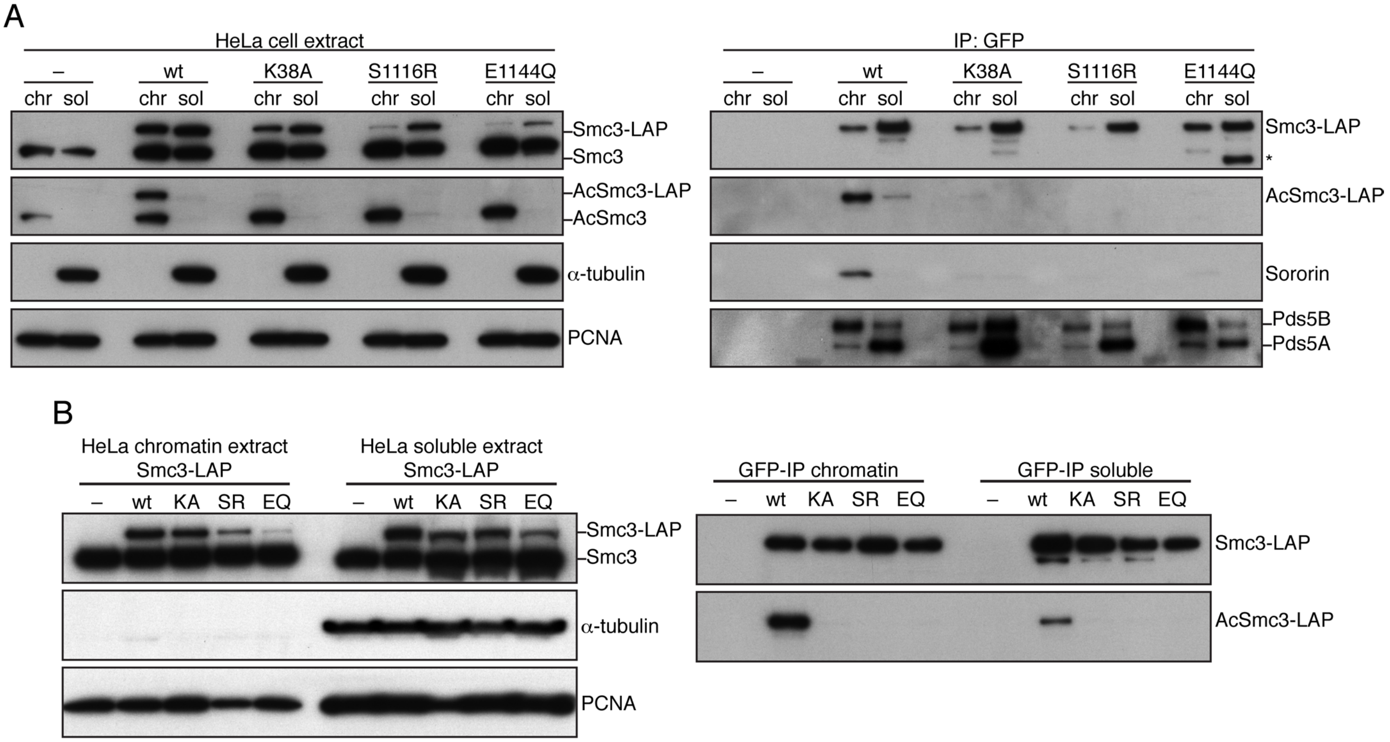

C

		Smc3-LAP			
		wild-type	K38A	S1116R	E1144Q
identified proteins	molecular weight	number of unique peptides identified			
Smc3	142 kDa	117	79	120	111
Smc1A	143 kDa	107	85	124	109
Pds5B	165 kDa	84	52	84	82
SA2	146 kDa	75	43	76	67
Wapal	143 kDa	66	45	65	60
Pds5A	151 kDa	64	22	58	46
SA1	144 kDa	52	52	55	46
Rad21	72 kDa	45	34	46	41
Sororin	28 kDa	14	5	4	4
Esco2	93 kDa	2	0	0	0

Figure S2, related to Figure 2. Cohesin ATPase mutants associate transiently with chromatin

(A) HeLa cells containing Smc3-LAP wild-type and mutants were grown asynchronously and Smc3-LAP was immunoprecipitated from soluble (sol) and nuclease-treated chromatin (chr) fractions as indicated. HeLa cells without Smc3-LAP were used as control. Asterisk denotes cross-reacting protein band.
(B) Cells were synchronized in G2-phase and soluble and nuclease-treated chromatin fractions were prepared and immunoprecipitated as indicated.
(C) Smc3-LAP immunoprecipitation from nuclease-digested chromatin fractions was subjected to MS analysis.

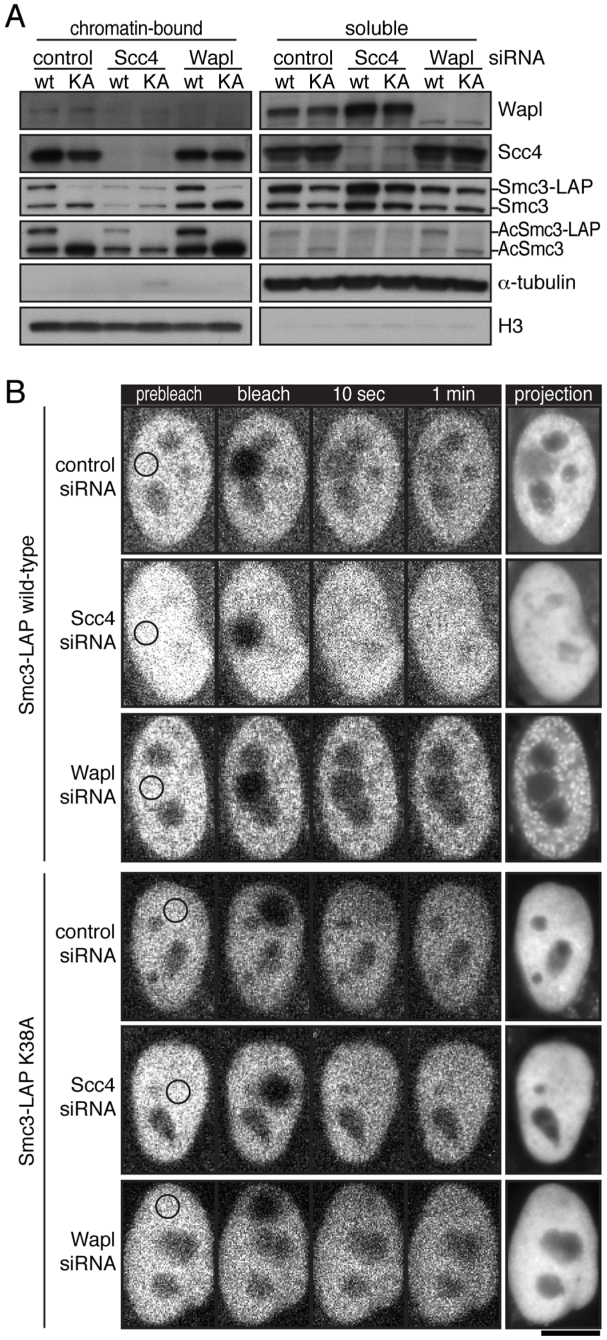

Figure S3, related to Figure 3. Chromatin association of cohesin ATPase mutants depends on Scc4, but not on Wapl

(A) Chromatin and soluble fractions of cells from Figure 3D,E were analyzed by immunoblotting to verify efficient protein depletion. Please note that the chromatin-bound KA mutant extract after Scc4 RNAi contained some α-tubulin, indicating the presence of some soluble material. This might explain why more Smc3-LAP KA is detected on chromatin after Scc4 depletion (lane 4) than after control depletion (lane 2).

(B) Still images of FRAP experiments in Figure 3D,E showing fluorescence recovery at defined time points and image projections of all 520 time frames ('sum slices'). Note that wild-type Smc3-LAP but not Smc3-LAP KA is seen in speckled structures after depletion of Wapl. These structures might represent chromatin regions in which cohesin is stably bound, similar to how cohesin is seen in axial structures ('vermicelli') in *Wapl*$^{-/\Delta}$ mouse embryonic fibroblasts [S1]. The observation that Smc3-LAP KA is not enriched in such speckles is consistent with the notion that this mutant cannot be stabilized on chromatin by Wapl depletion. Scale bar represents 10 μm.

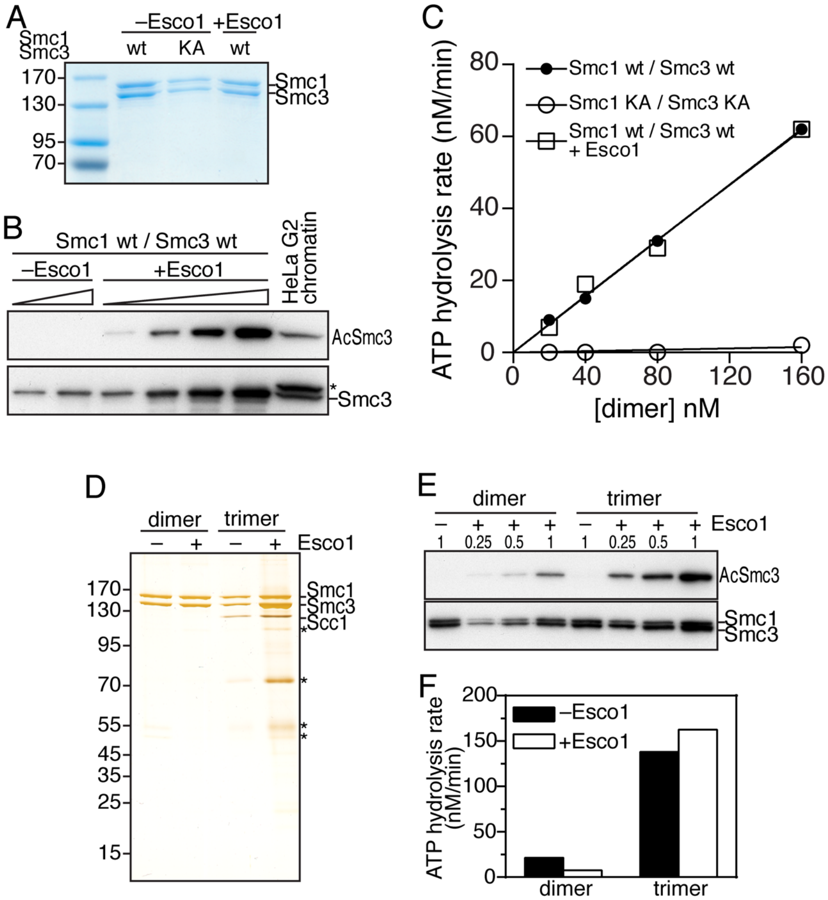

Figure S4, related to Figure 4. Smc3 acetylation does not detectably affect cohesin's ATPase activity

(A) Coomassie staining of cohesin dimers expressed in the presence or absence of human Esco1 and purified from insect cells.

(B) Cohesin complexes from (A) were immunoblotted for Smc3 and acetylated Smc3. Increasing amounts of purified proteins were loaded and compared to acetylation levels of fractionated chromatin from HeLa cells synchronized in G2-phase. Asterisk denotes cross-reacting protein band. Note that co-expression of cohesin dimers with Esco1 results in levels of Smc3 acetylation that are comparable to the levels of Smc3 acetylation that are observed in chromatin fractions of HeLa cells synchronized in G2-phase. Because in G2-phase about half of all chromatin bound cohesin complexes are stably bound [S2] and these are thought to be stabilized by sororin [S3] which only binds to acetylated cohesin [S4, S5], these results imply that in the sample containing recombinant cohesin dimer approximately half of the molecules were acetylated.

(C) Quantification of ATP hydrolysis rate from samples in (A) as a function of enzyme concentration.

(D) Silver staining of cohesin complexes expressed with or without Esco1. Asterisks denote unidentified proteins.

(E) Cohesin complexes from (D) were immunoblotted for Smc1, Smc3 and acetylated Smc3. Increasing amounts of acetylated extracts were loaded to compare acetylation levels between dimeric and trimeric complexes.

(F) Quantification of ATP hydrolysis of cohesin expressed in the presence or absence of Esco1.

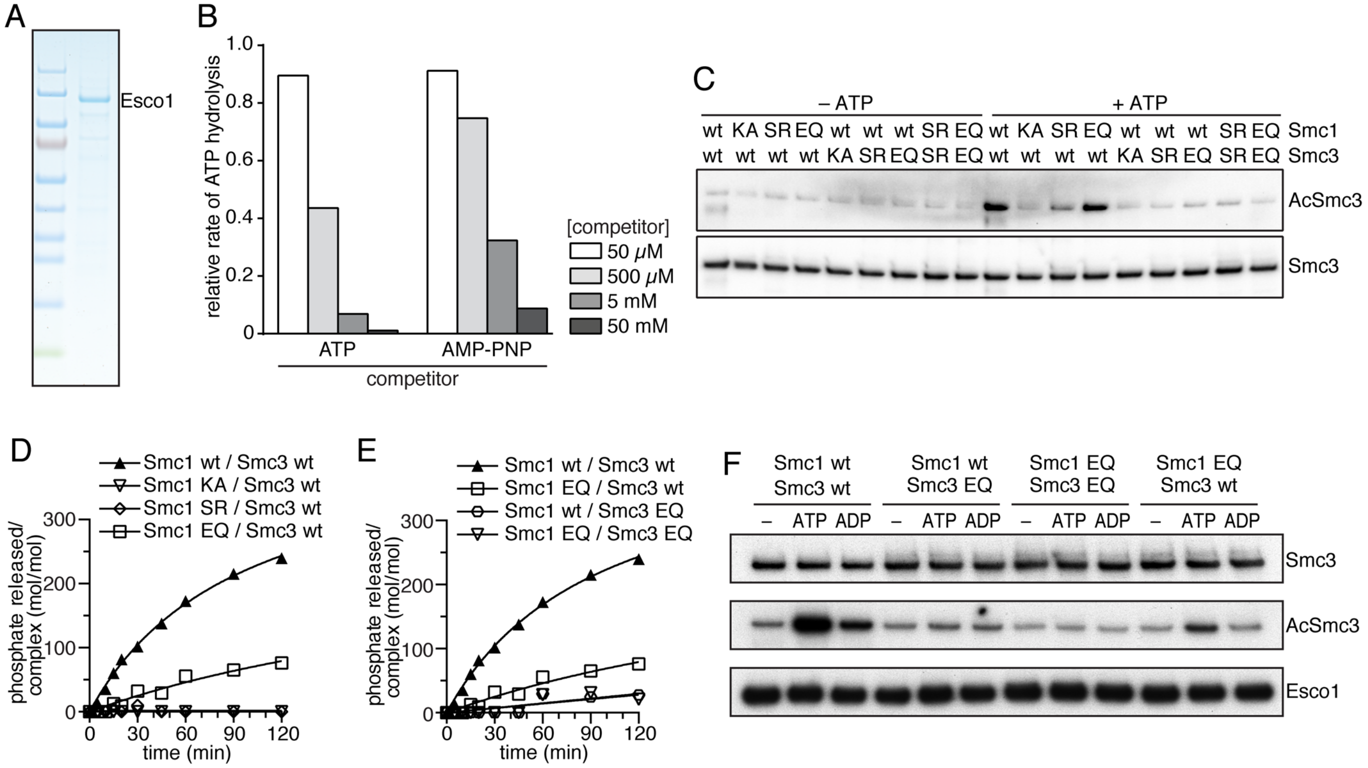

Figure S5, related to Figure 5. Cohesin's nucleotide-bound state is important for Smc3 acetylation.

(A) Purified human Esco1 was analyzed by Coomassie staining.
(B) Quantification of relative ATP hydrolysis rates after supplementing a reaction containing 50 μM ATP and 12.5 nM γ-ATP with different concentrations of competitor.
(C) Different mutant cohesin trimers were subjected to the acetylation reaction in the absence or presence of ATP.
(D) Time course quantification of phosphate released from ATPase reactions with trimeric cohesin containing Smc1 mutations.
(E) Time course quantification of phosphate released from ATPase reactions with Walker B mutation-containing cohesin trimers.
(F) Wild-type and Walker B mutant complexes were subjected to the acetylation reaction in the presence of ATP or ADP. Note that Smc3 acetylation was not increased by ADP if the Walker B motif of Smc1 and/or Smc3 had been mutated (Smc1 EQ, Smc3 EQ), even though these mutants are predicted to be able to bind ADP. This implies either that ADP binding is not sufficient for Smc3 acetylation, or that these mutants cannot bind ADP as well as is predicted.

Supplemental Experimental Procedures

Antibodies. Antibodies against the following proteins were described previously: SA1 (A823); SA1/SA2 (A786); Smc3 (A845, human; A846, *Xenopus*) [S6]; Scc4 (A974, [S7]); Wapl (A961, [S8]); Sororin (A953, [S3]); Esco1 (A782); Scc1 (A900); acetyl-Smc3 (A683, [S5]). Polyclonal rabbit anti-*Xenopus* XCAP-E and Orc2 antisera were kindly provided by E. Watrin and J.J. Blow respectively [S9, S10]. Goat anti-GFP antibody for immunoprecipitation was a kind gift from A.A. Hyman.

The following commercial antibodies were used: Anti-GFP antibody (Abcam, ab13970); Smc1 antibody (Bethyl laboratories, A300-55A); PCNA antibody (Santa Cruz, sc-56); histone H3 antibody (Cell Signaling, 9715L or Santa Cruz, sc-8654); Scc2 antibody (Absea, 010702F01 and 010516H10).

Plasmids and Proteins. Human Smc1-His_6, Smc3-FLAG and HA-Scc1 were cloned into pFastbac1 (Invitrogen) and Esco1 was cloned in frame with the His_6 in pFastbac-HTC (Invitrogen). Point mutations in Smc1 and Smc3 were introduced by PCR. Bacmids and Baculoviruses were generated and used for protein expression in Sf9 insect cells according to Invitrogen's bac-to-bac manual.

Dimers were expressed by coinfection of Smc1-His_6 and Smc3-FLAG viruses. In order to express tetrameric complexes, Smc1, Smc3-FLAG, Scc1 and His_{10}-SA1 were combined on a pFL multibac vector [S11]. Trimeric cohesin complexes used in Figure 1 were expressed by combining Smc1, Smc3-FLAG, Scc1-His_{10} on a pFL multibac vector whereas trimers used in subsequent figures were expressed in Sf9 insect cells by co-infection. Human Esco1 was cloned in frame with the His_6 tag in pFastbac-HTC for protein expression.

Frozen cell pellets were dounce-homogenized in 2.5 pellet volumes of buffer A (25 mM Tris pH 8.0, 100 mM NaCl, 10% glycerol) supplemented with 5 mM PMSF and 0.5% Tween-20. Lysates were clarified by centrifugation using a 70Ti rotor for 1 hour at 45,000 rpm and incubated with anti-FLAG M2 beads (Sigma) or with 1 mg ml^{-1} anti-HA (12CA5) bound to Bio-Rad Protein A agarose beads for 2 hours to purify dimers and trimers respectively. Bound proteins were washed three times with 10 bead volumes of buffer A containing 0.05% Tween-20 and eluted three times using one bead volume FLAG or HA peptide (0.1 mg ml^{-1}). The eluates were supplemented with 15 mM imidazole and incubated with Ni-NTA beads (Qiagen). Bound proteins were washed with buffer B (150 mM NaCl, 25 mM sodium phosphate pH 7.5, 10% glycerol, 20 mM β-mercaptoethanol, 15 mM imidazole) and eluted with buffer B containing 500 mM imidazole. Proteins were dialyzed against buffer A (slide-a-lyzer dialysis cassette; Pierce 66110), aliquoted and snap-frozen.

Esco1 was expressed in Sf9 cells, purified using buffer B supplemented with 0.5% Tween-20 and 5 mM PMSF and processed as described above.

Sea urchin Δ90 cyclin B was prepared as described [S12].

ATPase assays. Cohesin complexes were incubated in 10 μl reactions containing buffer A with 0.1 mg ml^{-1} BSA, 1 mM $MgCl_2$, 10 nM [γ-^{32}P] ATP and 50 μM cold ATP unless stated otherwise. Reactions were incubated at 37°C and stopped by adding 1% SDS and 10 mM EDTA at the indicated time points. One μl of the reaction was spotted on a polyethyleneimide plate (EMD Biosciences) and the reaction products were separated by TLC using 0.75 M KH_2PO_4 (pH 3.4). The plates were dried and analyzed using a phosphorimager (Bio-Rad). The relative levels of P_i were used as a measure of the ATPase activity.

Acetylation Assays. Acetylation assays were performed in 10 μl reactions containing buffer A supplemented with 0.05 mg ml^{-1} BSA, 1 mM $MgCl_2$, 10 μM acetyl-CoA, 50 nM Esco1 and 150 nM cohesin complex. Reactions were performed in the absence or presence of 50 μM ATP, ADP or AMP-PNP at 37 °C for 1 hr and stopped by adding Laemmli buffer. Antibody A683 after immunoblotting was used to detect the acetylation of Smc3.

Animal experimentation. Experiments on living animals were approved by the IMP IMBA Institutional Animal Welfare Board in accordance with the Austrian Animal Experiments Act of 2012 and Directive 2010/63/EU.

***Xenopus* Egg Extract Preparation and Immunodepletion.** *Xenopus* egg extracts were prepared as described [S13]. Cycloheximide (250 μg ml^{-1}), 25 mM phosphocreatine, 15 μg ml^{-1} creatine phosphokinase, and 0.3 mM $CaCl_2$ were added to extract before use.

Demembranated *Xenopus* sperm nuclei were prepared as described previously [S14]. Sperm was added at 3333 nuclei per μl extract for chromatin isolation experiments and 1111 nuclei per μl extract for immunofluorescence microscopy of *Xenopus* chromosomes. Following sperm addition, extracts were incubated at 23 °C for 2 hours to allow completion of DNA replication and then either driven into mitosis or processed for chromatin isolation. To induce mitosis, nondegradable Δ90 cyclin B was added to egg extract at 300 nM and incubated for a further 3 hours prior to mitotic chromosome isolation and immunofluorescence analysis.

For immunodepletion of cohesin from *Xenopus* egg extracts, two rounds of immunodepletion were performed using an anti-SA1 antibody (A823) followed by a single round using an anti-SA1/SA2 antibody (A786). Affinity-purified antibodies were conjugated to Affi-Prep Protein A Matrix (Bio-Rad) at a ratio of 8.3 μg antibody per μl of beads. Beads were added to interphase

extracts (15 μl beads per 100 μl extract) and incubated on a rotator for 40 min at 4 ^{o}C. Beads were removed by centrifugation.

Preparation of *Xenopus* Chromatin Fractions. Extract was diluted 25-fold in ice cold nuclear isolation buffer (50 mM HEPES-KOH pH 7.6, 50 mM KCl, 5 mM $MgCl_2$, 2 mM DTT, 0.5 mM spermidine, 0.15 mM spermine and 1:1000 protease inhibitor mix) supplemented with 0.1% Triton X-100 and underlayed with 100 μl of this buffer plus 15% sucrose. Samples were centrifuged in a swinging bucket rotor (2100 g, 5 min, 4 ^{o}C) and the pellet recentrifuged in a fixed-angle rotor (15000 g, 2 min, 4 ^{o}C) and resuspended in SDS sample buffer.

Immunofluorescence Microscopy of *Xenopus* Chromosomes. For XCAP-E staining of mitotic *Xenopus* chromosomes, extracts were fixed for 15 min at room temperature with 50 volumes of XBE2 (10 mM HEPES pH 7.7, 100 mM KCl, 2 mM $MgCl_2$, 0.1 mM $CaCl_2$, 5 mM EGTA, 50 mM sucrose) supplemented with 2% formaldehyde and 0.25% Triton X-100. The specimens were layered on top of 30% glycerol in XBE2 and spun onto poly-Lysine-coated coverslips at 3000 g for 15 min. Coverslips were then incubated with 0.5% Triton X-100 in PBS for 5 min, blocked with 3% BSA in PBS, and incubated with anti-XCAP-E antibody.

BAC mutagenesis, HeLa cell culture, RNA interference and FACS. Mouse Smc3-LAP bacterial artificial chromosome mutants were generated as described previously [S5] and transfected into HeLa Kyoto cells. siRNA duplexes were pre-mixed with RNAiMax (Invitrogen) according to manufacturer's instructions, added directly to cells at final concentrations of 30 nM and incubated for 48 or 72 hours. For G1-phase synchronization, cells were treated with 2 mM thymidine for 24 hours and released into fresh medium for 15 hours. Cells for FRAP experiments were grown in 8-well Labtek II chambered coverglass (Nunc). Fifteen minutes

prior to imaging, Hoechst was added at a final concentration of 0.2 µg ml^{-1} to visualize DNA and cycloheximide was used at 1 µg ml^{-1} to inhibit protein synthesis.

Synthetic siRNA oligonucleotides were purchased from Ambion. Sense sequences of siRNA oligos are: Scc4 GAAUUGUACUGUCAAGAGAtt; Wapl CGGACUACCCUUAGCACAAtt. siRNA against firefly GL2 luciferase was used as a control [S15].

Cell-cycle profiling was performed using propidium iodide staining as previously described [S16] with minor modifications. In brief, cells were resuspended in 0.8 ml PBS and fixed with 2.2 ml ice-cold methanol. Cells were washed with PBS, resuspended in PI buffer (50 μg ml^{-1} propidium iodide, 10 mM Tris pH 7.5, 5 mM $MgCl_2$, 200 μg ml^{-1} RNase A) and analyzed using a FACS Canto flow cytometer and FACSDiva software (BD Biosciences).

HeLa cell extracts, immunoblotting and immunoprecipitation. Cell pellets were resuspended in extraction buffer (25 mM Tris pH 7.5, 100 mM NaCl, 5 mM $MgCl_2$, 0.2% NP-40, 10% glycerol, 10 mM sodium butyrate, ‘complete’ protease inhibitor mix (Roche)) and lysed on ice by passing through a hypodermic needle. To fractionate soluble and chromatin-bound proteins, the homogenate was spun at 1300 *g* and washed three times with extraction buffer. Pellets were resuspended in Laemmli’s sample buffer, heated to 95 °C, and passed over a 0.45 μm filter. Immunoblotting was performed as described [S7]. To release proteins from chromatin, samples were treated with benzonase (250 U ml^{-1}) and insoluble material was removed by centrifugation. Supernatant extract was concentration adjusted, added to crosslinked antibody beads, incubated, washed, and eluted with 0.1 M glycine pH 2.0. To analyze interacting proteins by mass spectrometry as described in Figure S2, cells were synchronized in G2-phase by a 6 h release from thymidine arrest and chromatin extracts were

prepared as described above with a single washing step. Immunoprecipitation was performed using crosslinked anti-GFP antibody beads, and eluates were processed for in-solution digest and mass spectrometry as described [S17].

HeLa cell immunofluorescence microscopy. Cells grown on coverslips were pre-extracted with 0.1% Triton X-100 for 2 min as described previously [S18], PBS-washed and fixed with 4% paraformaldehyde in PBS. After fixation, cells were permeabilized with 0.1% Triton X-100 in PBS for 5 min, blocked with 3% BSA in PBS containing 0.01% Triton X-100 and incubated with primary and secondary antibodies (Alexa Fluor 488, Alexa Fluor 568 and Alexa Fluor 647; Molecular Probes). DNA was counterstained with DAPI. Coverslips were mounted onto slides with ProLong Gold (Molecular Probes).

Images were taken on a Zeiss Axioplan 2 microscope with 63x Plan-Apochromat objective lense (Zeiss). The system was equipped with a CoolSnapHQ CCD camera (Photometrics).

Fluorescence recovery after photobleaching (FRAP). Cells synchronized in G1-phase were imaged on an LSM5 Duo (Zeiss) confocal microscope using a 63x Plan-Apochromat objective and open pinhole. Twenty images were acquired before bleaching a radial spot (r = 2 µm) three times at 100% laser intensity (100 mW diode 488). Five hundred images were acquired afterwards at 200 ms intervals. Signal intensities were measured using ImageJ at bleached, nuclear and background regions and normalized according to Ellenberg *et al.* [S19]. Data was analyzed using Berkeley Madonna and a sum of three exponential functions that represent nuclear diffusing, transiently chromatin associated and chromatin bound populations a=ini*((1-(perctrans+percstabl))*(1-EXP(-(kOffdiff)*time))+perctrans*(1-EXP(-(kOfftrans)*time))

+percstabl*(1-EXP(-(kOffstabl)*time))). The diffusion parameter D was measured using the formula $I(t) = I_{final} * (1 - sqrt(4r^2/(4r^2+4*\pi*D*t)))$.

Data quantification and analysis. Quantifications were processed with Microsoft Excel 2007 and GraphPad Prism 6.

Supplemental References

S1. Tedeschi, A., Wutz, G., Huet, S., Jaritz, M., Wuensche, A., Schirghuber, E., Davidson, I.F., Tang, W., Cisneros, D.A., Bhaskara, V., *et al.* (2013). Wapl is an essential regulator of chromatin structure and chromosome segregation. Nature *501*, 564-568.

S2. Gerlich, D., Koch, B., Dupeux, F., Peters, J.M., and Ellenberg, J. (2006). Live-cell imaging reveals a stable cohesin-chromatin interaction after but not before DNA replication. Curr. Biol. *16*, 1571-1578.

S3. Schmitz, J., Watrin, E., Lenart, P., Mechtler, K., and Peters, J.M. (2007). Sororin is required for stable binding of cohesin to chromatin and for sister chromatid cohesion in interphase. Curr. Biol. *17*, 630-636.

S4. Lafont, A.L., Song, J., and Rankin, S. (2010). Sororin cooperates with the acetyltransferase Eco2 to ensure DNA replication-dependent sister chromatid cohesion. Proc. Natl. Acad. Sci. U. S. A. *107*, 20364-20369.

S5. Nishiyama, T., Ladurner, R., Schmitz, J., Kreidl, E., Schleiffer, A., Bhaskara, V., Bando, M., Shirahige, K., Hyman, A.A., Mechtler, K., *et al.* (2010). Sororin mediates sister chromatid cohesion by antagonizing Wapl. Cell *143*, 737-749.

S6. Sumara, I., Vorlaufer, E., Gieffers, C., Peters, B.H., and Peters, J.M. (2000). Characterization of vertebrate cohesin complexes and their regulation in prophase. J. Cell Biol. *151*, 749-762.

S7. Watrin, E., Schleiffer, A., Tanaka, K., Eisenhaber, F., Nasmyth, K., and Peters, J.M. (2006). Human Scc4 is required for cohesin binding to chromatin, sister-chromatid cohesion, and mitotic progression. Curr. Biol. *16*, 863-874.

S8. Kueng, S., Hegemann, B., Peters, B.H., Lipp, J.J., Schleiffer, A., Mechtler, K., and Peters, J.M. (2006). Wapl controls the dynamic association of cohesin with chromatin. Cell *127*, 955-967.

S9. Oehlmann, M., Score, A.J., and Blow, J.J. (2004). The role of Cdc6 in ensuring complete genome licensing and S phase checkpoint activation. J. Cell Biol. *165*, 181-190.

S10. Watrin, E., Cubizolles, F., Osborne, H.B., Le Guellec, K., and Legagneux, V. (2003). Expression and functional dynamics of the XCAP-D2 condensin subunit in Xenopus laevis oocytes. J. Biol. Chem. *278*, 25708-25715.

S11. Fitzgerald, D.J., Berger, P., Schaffitzel, C., Yamada, K., Richmond, T.J., and Berger, I. (2006). Protein complex expression by using multigene baculoviral vectors. Nat Methods *3*, 1021-1032.

S12. Glotzer, M., Murray, A.W., and Kirschner, M.W. (1991). Cyclin is degraded by the ubiquitin pathway. Nature *349*, 132-138.

S13. Gillespie, P.J., Gambus, A., and Blow, J.J. (2012). Preparation and use of Xenopus egg extracts to study DNA replication and chromatin associated proteins. Methods *57*, 203-213.

S14. Lebofsky, R., Takahashi, T., and Walter, J.C. (2009). DNA replication in nucleus-free Xenopus egg extracts. Methods Mol. Biol. *521*, 229-252.

S15. Elbashir, S.M., Harborth, J., Lendeckel, W., Yalcin, A., Weber, K., and Tuschl, T. (2001). Duplexes of 21-nucleotide RNAs mediate RNA interference in cultured mammalian cells. Nature *411*, 494-498.

S16. Wendt, K.S., Yoshida, K., Itoh, T., Bando, M., Koch, B., Schirghuber, E., Tsutsumi, S., Nagae, G., Ishihara, K., Mishiro, T., *et al.* (2008). Cohesin mediates transcriptional insulation by CCCTC-binding factor. Nature *451*, 796-801.

S17. Gregan, J., Riedel, C.G., Petronczki, M., Cipak, L., Rumpf, C., Poser, I., Buchholz, F., Mechtler, K., and Nasmyth, K. (2007). Tandem affinity purification of functional TAP-tagged proteins from human cells. Nat. Protoc. *2*, 1145-1151.

S18. Hauf, S., Roitinger, E., Koch, B., Dittrich, C.M., Mechtler, K., and Peters, J.M. (2005). Dissociation of cohesin from chromosome arms and loss of arm cohesion during early mitosis depends on phosphorylation of SA2. PLoS Biol. *3*, e69.

S19. Ellenberg, J., Siggia, E.D., Moreira, J.E., Smith, C.L., Presley, J.F., Worman, H.J., and Lippincott-Schwartz, J. (1997). Nuclear membrane dynamics and reassembly in living cells: targeting of an inner nuclear membrane protein in interphase and mitosis. J. Cell Biol. *138*, 1193-1206.
